# Evidence for sparse synergies in grasping actions

**DOI:** 10.1038/s41598-017-18776-y

**Published:** 2018-01-12

**Authors:** Roberto Prevete, Francesco Donnarumma, Andrea d’Avella, Giovanni Pezzulo

**Affiliations:** 1 Department of Electric Engineering and Information Technologies (DIETI) Università di Napoli Federico II, Naples, Italy; 2 Institute of Cognitive Sciences and Technologies, National Research Council (ISTC-CNR), Via S. Martino della Battaglia, 44, 00185 Rome, Italy; 3 Department of Biomedical and Dental Sciences and Morphofunctional Imaging, University of Messina, Messina, Italy; 4 Laboratory of Neuromotor Physiology, Santa Lucia Foundation, Rome, Italy

## Abstract

Converging evidence shows that hand-actions are controlled at the level of synergies and not single muscles. One intriguing aspect of synergy-based action-representation is that it may be intrinsically sparse and the same synergies can be shared across several distinct types of hand-actions. Here, adopting a normative angle, we consider three hypotheses for hand-action optimal-control: *sparse-combination hypothesis (SC)* – sparsity in the mapping between synergies and actions - i.e., actions implemented using a sparse combination of synergies; *sparse-elements hypothesis (SE)* – sparsity in synergy representation – i.e., the mapping between degrees-of-freedom (DoF) and synergies is sparse; *double-sparsity hypothesis (DS)* – a novel view combining both SC and SE – i.e., both the mapping between DoF and synergies and between synergies and actions are sparse, each action implementing a sparse combination of synergies (as in SC), each using a limited set of DoFs (as in SE). We evaluate these hypotheses using hand kinematic data from six human subjects performing nine different types of reach-to-grasp actions. Our results support DS, suggesting that the best action representation is based on a relatively large set of synergies, each involving a reduced number of degrees-of-freedom, and that distinct sets of synergies may be involved in distinct tasks.

## Introduction

Understanding how the brain represents and controls human movements is a key challenge in computational motor control^1–3^. To generate meaningful movements, the Central Nervous System (CNS) has to coordinate the numerous Degrees of Freedom (DoF) of the musculoskeletal system. Selecting the appropriate DoF patterns to achieve a purposeful movement is extremely demanding task given the huge dimensionality of the search space and nonlinearities in it. One paradigmatic example is the control of the human hand: a very complex system with many degrees of freedom that allows us to dexterously perform complex actions such as grasping an apple or twisting and detaching its stem.

Several studies have highlighted that motor control at the level of single muscles is unlikely, arguing in favor of a simplified way of controlling actions, such as for example hand grasping actions^4–7^–with the most well-studied example being the concept of *synergies*^8–11^. Broadly speaking, synergies refer to specific patterns of muscular activity or movement kinematics/dynamics, which can be used by the central nervous system (CNS) as building blocks to represent and control actions, thus alleviating the problem of controlling a large number of muscles. However, different view on synergies have been proposed, which range from kinematic and dynamic synergies^8,12,13^ to postural^5,6,14^ and temporal postural synergies^9,15,16^. Given this proliferation of definitions and conceptualizations of synergies, some fundamental questions remain open concerning the very nature of synergy-based coding^10,17,18^.

One intriguing aspect of synergy-based coding is that it may be intrinsically sparse and the same synergies can be shared across several distinct hand actions. Several studies showed that a reduced number of hand synergies explains much of the variance of the hand-shape during reach-to-grasp actions irrespectively of what is the hand action type^15,19^. For example in^15^ two hand synergies account for about 80% of the variability of the hand shapes at the end of the reach. Still, it is unclear at what level synergy-based coding is sparse.

**Figure 1:**
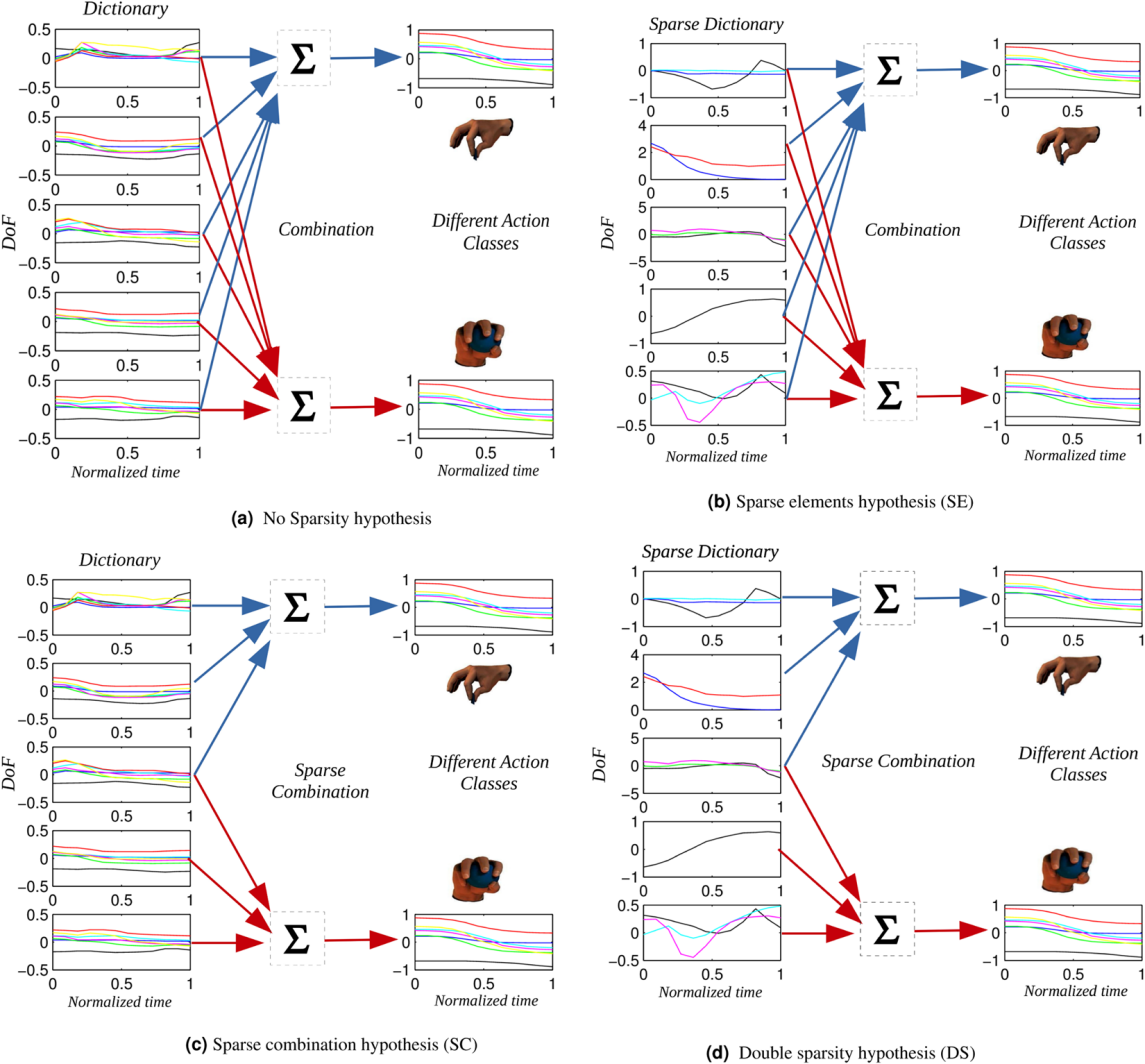
Hypotheses on Synergy recombinations. Panel (a) shows no sparsity in the combination of synergies. This can be considered the “null hypothesis”. Panel (b) shows the Sparse Elements hypothesis (SE). It shows an example of a predefined set of five Temporal Postural Synergies, all of which are implied in both action classes. However, each synergy only involves a restricted number of Degrees-of-Freedom. Panel (c) shows the Sparse Combination hypothesis (SC). Given a large enough set of predefined synergies, different actions are represented by different subsets of the predefined synergies, which can overlap. The Figure shows an example of a predefined set of five Temporal Postural Synergies, where the first two synergies are used to represent actions belonging to one action class, the last two synergies are used to represent actions belonging to another action class, and the third synergy is used to represent both action classes. Each synergy involves all the Degrees-of-Freedom (six in this example). Panel (d) shows the Double Sparsity hypothesis (DS). It shows an example of a predefined set of five Temporal Postural Synergies, where the first two synergies are used to represent actions belonging to one action class, the last two synergies are used to represent actions belonging to another action class, and the third synergy is used to represent both action classes. Furthermore, each synergy only involves a restricted number of Degrees-of-Freedom. See main text for more details.

In this article we directly compare three competing hypotheses on sparse coding of synergies from a normative perspective, by asking which one is more appropriate for the (optimal) control of hand movement. According to the *sparse combination hypothesis (SC)* (Fig. 1c)), each action is represented by a *sparse combination* of the predefined synergies. Accordingly, it exists a relatively large set of synergies, and a subset of them can be used (and shared) across many specific tasks^16,19–25^. In^16,19^, the authors highlight that specific types of actions use separate synergy sub-spaces that can overlap, within the space defined by the synergies. The results reported in^21,22^ suggest the presence of a mixture of synergies shared across behaviors as well as synergies for specific behaviors. In^20,25^ it is suggested that the CNS switches between different sets of synergies for distinct grasping and manipulation tasks. If this hypothesis holds, given a large enough set of predefined synergies, different actions should be represented by different subsets of the predefined synergies which can overlap.

According to the *sparse elements hypothesis (SE)* (Fig. 1b), each synergy is sparse in that it only uses a limited set of DoF or controls a limited set of muscles (possibly overlapping between synergies); however, unlike SC, actions use a mixture of all available synergies, not a subset. Supporting this claim is the presence of brain mechanisms that contribute to the dynamic emergence of task-specific muscle synergies appropriate for a wide range of abstract task goals, with the possibility to have synergies including specific subsets of muscles according to abstract task demands^24^. A related concept in terms of muscle activity is expressed in^26^, where it is suggested that the detailed control of different muscle groups is necessary for the flexible control of multi-articular movements. Thus, one can hypothesize a neural control based on a relatively large set of synergies, each involving a restricted number of degrees-of-freedom and possibly overlapping among the different synergies.

However, none of the two hypotheses SE and SC can explain the whole range of experimental data reported above. For this, here we introduce and test a novel hypothesis, the *double sparsity hypothesis (DS)* (Fig. 1d), which combines the two previous hypotheses: it assumes that both the mapping between synergies and actions (as in SC) and the mapping between DoF/muscles and synergies (as in SE) are sparse.

## Results

The aim of this study is disentangling between different hypotheses on how sparsity may apply to synergy-based hand movement representation. To this aim, we first consider a (baseline) condition that implies *no* sparsity in hand synergies:

- According to this (*baseline*) or *standard action representation (SAR)* hypothesis, there is no sparsity in synergy-based coding. This hypothesis is implemented here using Principal Component Analysis (PCA)^27^, which results in action representations that use a linear combination of *all* the available synergies, which in turn use *all* the available DoF–thus implying no sparsity.

In the last decade, there has been a growing interest in alternative approaches to PCA^27^ including most prominently *dictionary learning* methods^28,29^ that are widely used in several domains such as neuroscience, geophysical seismic sounding, acoustics and brain-computer interface^28,30–33^. Dictionary learning methods achieve more powerful signal representations using *overcomplete* dictionaries including prototype signals, and are able to find the underlying structure of environmental signals^29,34–39^–all features that make them particularly well suited to implement the three competing hypotheses that we want to test.

Therefore, we used dictionary learning methods to implement the three aforementioned hypotheses that imply (different kinds of) sparsity of synergy-based coding:

- According to the *sparse combinations (SC)* hypothesis, hand actions are represented as sparse combinations of a predefined set of basis elements (atoms or dictionary elements). This hypothesis is implemented here using the dictionary learning algorithm ${\ell }_{1}$-regularized^40^.
- According to the *Sparse Elements (SE)* hypothesis, hand actions are represented as combinations of a predefined set of basis elements (atoms or dictionary elements), each involving a reduced number of DoF. This hypothesis is implemented here using the dictionary learning algorithm *SSPCA*^27,41^.
- According to the *Double Sparsity (DS)* hypothesis, hand actions are represented as sparse combinations of a predefined set of basis elements (atoms or dictionary elements), each involving a reduced number of DoF. This hypothesis is implemented here using the dictionary learning algorithm *SRSSD*^42^.

### Testbed

**Figure 2:**
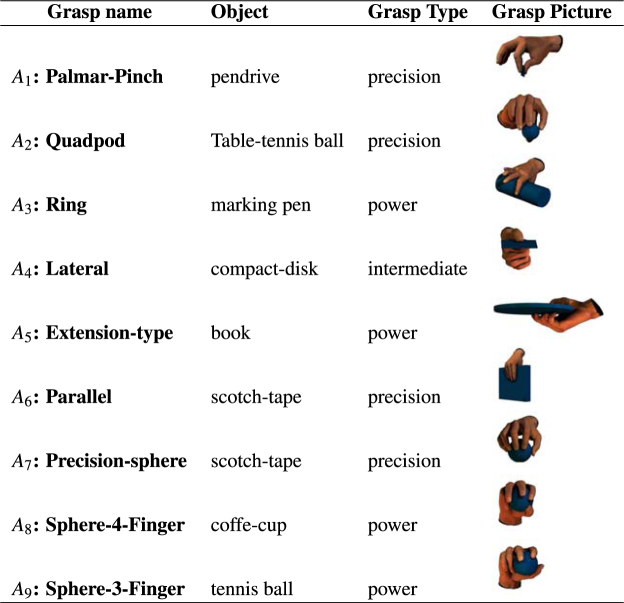
Grasping action types. The nine grasping action types *A*_1_, …, *A*_9_ used in our experiment. The grasp pictures are from http://http://grasp.xief.net and show the grasp types, see also^64,74^.

To obtain a testbed for this comparison, we collected data from six human subjects, each performing nine different class of reach-to-grasp actions (see Fig. 2), for a total of 450 grasping actions for each subject, using a dataglove (HumanGlove - Humanware S.r.l., Pontedera, Pisa, Italy) endowed with 16 hand-angle sensors. Note that datagloves have been successfully used for several studies on hand synergies^5,9,14,16^.

Using this testbed, we compared the action representations obtained under the four hypotheses (baseline with no sparsity, and three hypotheses with different kind of sparsity) in the context of action multi-class classification problems. The main criterion for the comparison is the classification accuracy of the four algorithms. This comparison will permit us to test for the improvements in performance due to sparsity at the level of the number of synergies and/or the subset of muscles represented within the various synergies–and ultimately determining at which level sparsity is optimal for the control of hand movements.

We considered two increasingly challenging experimental scenarios. In the *first scenario*, we considered the problem of classifying all the 9 action classes shown in Fig. 2. To test the robustness of the classification, we added to the recorded actions a zero-mean Gaussian noise, which results in (small) kinematic variations within the dataset. We expect the best data representations to exhibit more tolerance to kinematic noise than other representations.

In the *second scenario*, we considered permutations of the 9-classes original problem: we designed all the *c*-classes classification problems, with *c* = 2, 4, 6, 8, considering all the possible *c*-ples of action classes. This results into 255 distinct classification problems: 36 2-classes problems, 126 4-classes problems, 84 6-classes problems, and 9 8-classes problems. This second scenario permits to investigate the impact of sparsity when the number of classes increases.

Our experimental prediction is that sparse methods–and most prominently the method with double sparsity–should perform uniformly better than non-sparse methods like PCA, and this difference should increase with higher noise levels (showing robustness) and a greater number of action classes to be classified.

### First scenario: classification of 9 action classes

**Figure 3:**
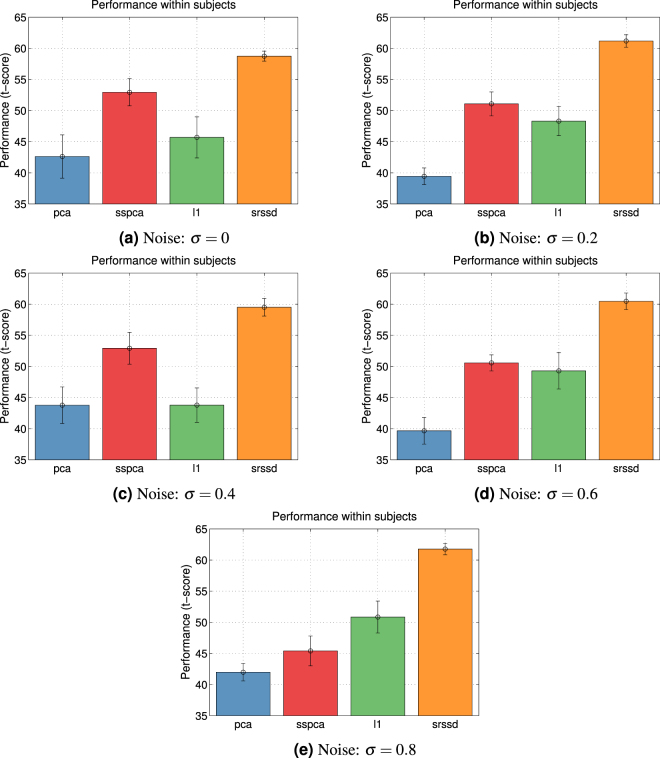
Accuracy considering all the classes. The Figure shows mean and standard deviation of the mean of the accuracies (t-score) computed on all the subjects in a multi-classes classification problem, including all the grasping classes, for the four approaches ${\ell }_{1}$, *SSPCA*, *SRSSD* and *PCA* (see Materials and Methods for further details). The accuracy (t-score) is plotted versus the five different Gaussian noises which we added to validation and test sets. Note that in the test phase the best performing dictionary size usually corresponds to the maximum dictionary size (24).

The *first scenario* consists in the classification of the 9 action classes shown in Fig. 2. Figure 3 shows the mean of the accuracies of the four approaches (*PCA*, ${\ell }_{1}$, *SSPCA*, and *SRSSD*), when considering data from all subjects. Each panel of the Fig. 3 shows accuracy plotted against the five different levels of Gaussian noise that we added to validation and test set (*σ* = 0, 0.2, 0.4, 0.6, 0.8).

In our comparisons, we considered the accuracy in classification of the best dictionary of each method, for each subject. We normalized the accuracy of each subject with respect to his or her overall performance, by considering a t-score of the overall subject’s accuracy distribution (mean and standard deviation), i.e., the subjects’ accuracy when considering all methods, dictionaries and trials (see Supplementary Materials for the raw accuracies). Then, we averaged the t-scores of all the subjects to obtain the accuracy of each method. We repeated the same procedure for both scenarios, separately. Note that these and all the following results are obtained on the test set; the parameters of the methods and of the classifier where those having the best performance on the validation set (see the Methods section for further details).

We performed five one-way analysis of variance (ANOVAs) to compare the accuracy of the four approaches (*PCA*, ${\ell }_{1}$, *SSPCA*, and *SRSSD*), with each of the five levels of noise (*σ* = 0, 0.2, 0.4, 0.6, 0.8). We found a significant effect of condition (approaches) in all the five cases. Post-hoc analyses (t-tests) showed that the *SRSSD* approach performs significantly better than all the other approaches, for all the five levels of noise (see Supplementary Materials).

In keeping with our experimental predictions, these results show that the three sparse methods perform generally better than *PCA*. *SSPCA* and *SRSSD* outperform *PCA* in all cases, whereas ${\ell }_{1}$ outperforms *PCA* in three out of five levels of noise. Importantly, the best performance is obtained using the *SRSSD* method that implements the double sparsity hypothesis. Furthermore, the results show that the higher the noise, the better the *SRSSD* performance compared to *PCA*.

**Figure 4:**
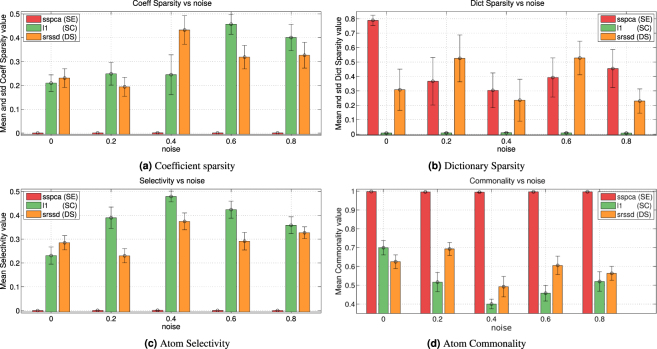
Sparsity, Selectivity and Commonality - first Scenario. Panel (a) shows mean and standard deviation of the mean of the coefficient sparsity in the linear combination of the dictionary elements, computed on all subjects. Panel (b) shows mean and standard deviation of the mean of the atom sparsity. Panel (c) shows mean and standard deviation of the mean of the atom selectivity. Panel (d) shows mean and standard deviation of the mean of the atom commonality.

As the three dictionary learning approaches have been run with sparsity parameters (*λ* and *eta*) that range within a set of different values, it is theoretically possible that the solutions selected for the classification problem do not actually result in sparse action representations, which would make these methods unfit to implement the SC, SE, and DS hypotheses. To rule out this possibility, Fig. 4a and b report the mean of the coefficient and dictionary sparsity versus noise for each approach (see formulas 6 and 7 in the Methods). Figure 4a shows that for ${\ell }_{1}$ and *SRSSD* the coefficient sparsity is largely greater than zero (in keeping with the SC and DS hypotheses) and it tends to increase as the the noise increases. On the contrary, for *SSPCA* this value is uniformly near to zero (in keeping with the SE hypothesis). Figure 4b shows that for ${\ell }_{1}$ the dictionary sparsity is uniformly near to zero (in keeping with the SC hypothesis). By contrast, for *SSPCA* and *SRSSD*, dictionary sparsity is strongly grater than zero (in keeping with the SE and DS hypotheses), with some values exceeding 0.5. There is no apparent dependence of dictionary sparsity from noise. These results confirm that the three dictionary learning approaches ${\ell }_{1}$, *SSPCA*, and *SRSSD* result in action representations that genuinely characterize the SC, SE, and DS hypotheses, respectively.

Figure 4c and d report the mean values of *selectivity* and *commonality* of atoms, respectively. Here, selectivity is high when an atom is only used to classify a single action class, whereas commonality is high when the same atoms are shared across many action classes (see definitions 8 and 9 in the Methods). These analyses show that the action representations obtained in ${\ell }_{1}$ and *SRSSD* use some atoms to represent actions belonging to many action classes (i.e., high commonality values) and other atoms to represent actions belonging to a specific action class (i.e., selectivity values different from zero). For example, with noise *σ* = 0.4, slightly more than 50% of the atoms in these two methods are shared across all the actions, and slightly less than 50% for specific action classes. This suggests a hierarchical organization of the dictionary, with a subset of atoms that are always used and the remaining atoms that are dedicated to specific action classes^16^.

### Second scenario: classification of 2, 4, 6 and 8 action classes

The second scenario consists in the classification of 2, 4, 6 and 8 action classes, while considering all the possible permutations of the 9 action classes of the first scenario. This implies that for each *c* value (with *c* = 2, 4, 6, 8) we consider $$(\begin{array}{c}9\\ c\end{array})$$ distinct classification problems, for a total of 255.

**Figure 5:**
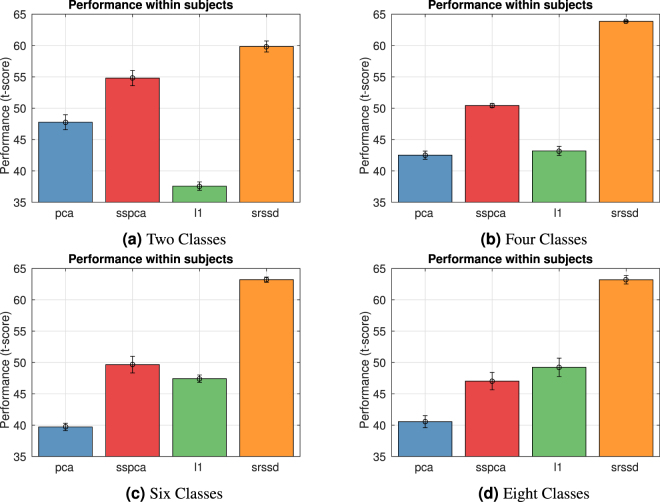
Accuracy wrt number of classes, second Scenario. The Figure shows mean and standard deviation of the mean of the accuracies (t-score) versus the number of classes of the *c*-classes classification problem (noise = *σ *= 0.8), computed on all subjects. Of note, for each value of *c* (see Supplementary Materials), we considered all the possible *c*-ple of class actions. Thus, for each *c* value we had $$(\begin{array}{c}9\\ c\end{array})$$ distinct classification problems.

Figure 5 shows the mean of the accuracies standardized across subjects using a t-score (calculated as for the First scenario, see Supplementary Materials for raw accuracies). The four panels show the classification results for the four different kinds of classification problems we considered (2, 4, 6 or 8 classes), with noise *σ* = 0.8.

We performed a one-way analyses of variance (ANOVAs) to compare the performance of the four approaches (*PCA*, ${\ell }_{1}$, *SSPCA*, and *SRSSD*), for each of the four different kind of classification problems. We found a significant effect of condition (approaches) in all the analyses. Post-hoc analyses (t-tests) showed that the *SRSSD* approach performs significantly better than all the other approaches, for all the four cases (see Supplementary Materials).

These results are in line with what reported in the First scenario: all the three sparse methods perform better than *PCA* and the greater the number of classes, the more the sparse methods outperform *PCA*–especially in the case of the *SRSSD* approach.

**Figure 6:**
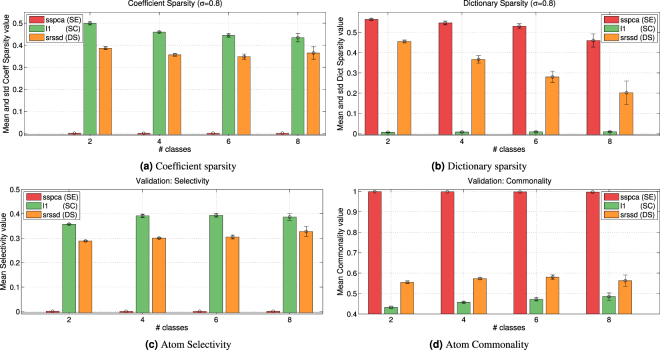
Sparsity, Selectivity and Commonality - second Scenario. Results computed on all subjects of the *c*-classes classification problem with noise *σ* = 0.8. Panel (a) shows mean and standard deviation of the mean of the coefficient sparsity versus the number of classes. Panel (b) shows mean and standard deviation of the mean of the atom sparsity. Panel (c) shows mean and standard deviation of the mean of the atom selectivity. Panel (d) shows mean and standard deviation of the mean of the atom commonality.

Figure 6a and b show the mean of the coefficient and dictionary sparsity, respectively, versus the number of classes, for each approach. Coefficient sparsity values measure how many elements of the dictionary (atoms) are used for representing the grasping actions (see formula (6)); whereas dictionary sparsity values measure how many DoFs are used to represent the grasping actions (see formula (7)). It emerges from Fig. 6a that for ${\ell }_{1}$ and *SRSSD* the coefficient sparsity is greater than zero and tends to remain unchanged, while for *SSPCA* it is uniformly zero, thus confirming the results in the first scenario. It emerges from Fig. 6b that, for *SSPCA* and (especially) *SRSSD*, dictionary sparsity remains greater than zero, coherent with the results of the first scenario. However, it also tends to decrease as the number of classes increases.

Figure 6c and d show the mean values of selectivity and commonality with respect to the number of action classes. Coherently with the results of the first scenario, the values of selectivity and commonality are uniformly higher than zero and tend to increase with the number of classes.

## Discussions

The way the human brain represents and controls action, and in particular hand actions such as grasping, is a widely discussed topic in neuroscience, sports science and rehabilitation^9,16,43,44^. While the importance of synergies for (hand) action control is well recognized^16,19,20,24,25^, some fundamental aspects of synergies-based control remain elusive. Contrasting theories propose sparsity in the mapping between synergies and actions, such that each action can be implemented based on a limited set of synergies (possibly shared across different actions) or in the mapping between degrees of freedom and synergies, such that each synergy can control a limited set of muscles.

In this paper, we addressed this question from a normative perspective, by asking at what level sparsity affords optimal control of movement. We compared three hypotheses on sparsity in synergy-based hand control. The *sparse combination hypothesis (SC)* assumes that actions are implemented based on a limited set of synergies, some of which are shared across actions. The *sparse elements hypothesis (SE)* assumes that synergies using only specific subsets of muscles according to the task. Given the limitations of these approaches in explaining the full range of available human data, we introduced a novel hypothesis–called the *double sparsity hypothesis (DS)*–which combines SC and SE and assumes sparsity in both the action-synergies and the synergies-DoF (or muscles) mappings. We implemented these three hypotheses using dictionary learning techniques (${\ell }_{1}$, *SSPCA*, and *SRSSD*) and compared them in a classification task based on a novel database of 450 human reach-to-grasp (hand) actions (see Fig. 2). As a baseline for the comparison, we used a (more traditional) *PCA* approach that corresponds to the hypothesis that there is no sparsity in in synergy-based hand control.

Our first and foremost criterion to compare the four approaches is classification accuracy under various levels of noise, which is an index of how “good” and robust the action representations developed under the different approaches are. To better understand the nature of the coding scheme afforded by the different dictionary learning approaches, we also analyzed the characteristics of the action representations they produce, including coefficient and dictionary sparsity as well as the *selectivity* and *commonality* of atoms, i.e., whether an atom is only used to classify a single action class (selectivity) or shared across many action classes (commonality).

Our results confirm that all the dictionary learning approaches outperform the more standard PCA method and are better able to classify–and find “good” representations–of the human hand actions. The results are coherent across the classification of 9 action classes (first scenario, see Fig. 3) or subsets of these classes (second scenario, see Fig. 5). The best results are obtained using the *SRSSD* approach, which implements the *double sparsity* hypothesis.

The classification results of the first scenario (Fig. 3) permit to appreciate that action representations obtained under the DS hypothesis (corresponding to *SRSSD* approach) are more noise tolerant than the other representations, suggesting that they may better capture its intrinsic and structural properties compared to alternative approaches. The classification results of the second scenario (Fig. 5) are coherent with this picture. Furthermore, they show that classification accuracy (especially under the DS hypothesis) tend to increase as the number of classes increases, coherent with our hypothesis that sparsity may be particularly beneficial when action classes are more numerous and the identification of their specific features is essential.

As a “safety check”, to confirm that our implementations are coherent with the hypotheses SC, SE and DS, we checked whether one can actually find sparsity in the action representations developed under the dictionary learning methods. Coherent with our interpretation, the results in the first scenario reveal sparsity of coefficients in *SRSSD* and ${\ell }_{1}$ but not *SSPCA* (Fig. 4a) and of dictionary in *SSPCA* and *SRSSD* but not ${\ell }_{1}$ (Fig. 4b). The results of the same analysis in the second scenario are largely consistent with this pattern (Fig. 6a and b), but also indicate that sparsity tends to decrease as the number of classes increases. This result is coherent with the observation that coefficient sparsity remains unchanged while the complexity of the data increases. As expected, for ${\ell }_{1}$ dictionary sparsity is zero.

Furthermore, we asked whether, using dictionary learning techniques, hand action classes actions can be represented using distinct, and possibly overlapping, sets of synergies. An analysis of *selectivity* (Fig. 4c) and *commonality* (Fig. 4d) in the first scenario show the significant presence of both selective and shared atoms. Even more tellingly, the results of the same analyses in the second scenario (Fig. 6c and d) show that selectivity and commonality values for the DS scheme tend to increases with the number of classes. These results are coherent with a composite representational scheme in which, as the number of classes increases, more atoms are required that generalize across them by representing their common features (commonality) but also more selective ones that permit distinguishing the actions belonging to different classes.

Taken together, these results show that dictionary learning methods that incorporate sparsity constraints outperform traditional methods to analyze synergies (*PCA*) with no sparsity. There is consensus in the literature that synergies simplify motor control by reducing the number of elements or degrees of freedom to be controlled. Our results show that a reduced number of parameters permits to reconstruct the whole set of grasping actions–and this “reduction” can take place in both the action-synergy and the DoF-synergy mappings. The best results are obtained in the presence of a double sparsity, i.e., sparsity in both the mapping between synergies and actions and the mapping between degrees of freedom (DoF) and synergies. In other words, classification accuracy is higher when hand action representations are based on a relatively large set of synergies involving a reduced number of degrees-of-freedom, and partially distinct sets of synergies are used for distinct tasks.

A potential limitation of this study is that it considers a limited number of object shapes (9 shapes) compared to other published works on kinematic hand synergies (e.g., 57 object shapes in^5^, 20 object shapes in^9^, and 50 object shapes in^23^). It thus remains to be fully assessed to what extent the results reported here generalize to more complex situations where many more objects are considered. In particular, a potential concern regards the extent to which the advantage of the double sparsity may derive from the structure of our specific data set. In principle, however, increasing the numer of objects might favor or disfavor sparsity depending on the distribution of object shapes and associated grasping action temporal profiles in the set. A large set of very similar object shapes might be captured by a sparser representation than a small set of very different shape. Testing the double sparsity hypothesis in larger data sets, or data sets having different statistical structure, remains an open objective for future research.

To what extent our results on classification accuracy can be used as an argument that the brain uses double sparsity for synergy coding? Our approach follows a long tradition of studying synergies using a computational approach that aims at modeling kinematic and/or dynamic data^5,6,8,9,16,19^. These studies have used methods like *PCA*^5,6^ to identify synergies in terms of small sets of elements that can be suitably combined to reconstruct the original kinematic and/or dynamic data. The key argument of this normative approach is that the Central Nervous System (CNS) faces a challenging problem–synergy control–that requires effective computational solutions. For this, computational algorithms that prove to be effective in (for example) classification might be taken as hypotheses on (or provide metaphors for) brain solutions to the same kind of problems. Convergent neurophysiological data^20,25,45,46^ supported these lines of argument in the case of synergies. Using the same normative approach as in these studies (and in many others in the fields of motor control and decision theory^47–49^) our results can be taken to suggest that the CNS might benefit from incorporating double sparsity constraints in synergy coding; or in other words, that the CNS should represent and control grasping actions by selecting a reduced number of atoms from a predefined dictionary of synergies, where each synergy involves a restricted set of DoFs.

Note that the task we use here–classification accuracy–may be particularly well suited to compare different schemes for action representation. This is because the ability of classification methods (including PCA and dictionary learning^50–52^) to discover semantic information derives in part from a notable property of the data: namely, the fact that data belonging to the same class can lie on or close to low-dimensional sub-spaces, sub-manifolds or stratifications–even if the original data are high dimensional, as in the kinematic/dynamic datasets used to study synergies. Thus, one should expect that the (kinematic/dynamic) data that are better classified are also the most meaningful for synergy-based coding, as they lie within relevant subspaces and exhibit more tolerance to kinematic noise than other representations.

Our computational analysis aligns well with an earlier analysis based on a musculoskeletal model^53^ and with empirical evidence^45,46,54–59^. Berniker and colleagues^53^, using model-order reduction techniques, identified a low-dimensional model of the non-linear dynamics of the frog’s limb and a set of muscle synergies effective at controlling it. While they did not explicitly enforce sparsity, the synergies that most compactly characterized the input–output dynamics were remarkably sparse (each synergy acting mainly on a particular joint of the limb). It is not clear whether the synergy combinations generated by an optimal controller performing reaching movements were also sparse but it would be interesting to test whether sparse synergy combinations improve the performance of a low-dimensional controller. Empirical evidence for a neural organization of synergies based on stimulation and recordings has been accumulating in recent years. Chemical stimulation of the interneurons in the frog’s spinal cord reveals the existence of a small set of muscle synergies^54^ with a topographic organization suggesting that a rostrocaudally travelling wave of activation underlies the control of locomotion^58^. In the mouse, optical stimulation shows that a specific population of molecularly identified pre-motor interneurons in the spinal cord encode muscle synergies^46^. Recordings from the spinal cord in frogs^55^ and monkeys^45,59^ also support the role of pre-motor interneurons in the organization of muscle synergies. In most cases the synergies organized by spinal pre-motor interneurons appear to involve a small number of muscles, supporting the neural organization of sparse modular elements. However, sparseness in the combinations is more difficult to assess experimentally because it requires testing a large number of conditions corresponding to different synergy combinations. Moreover, while muscle synergies may be organized by interneurons in the spinal cord and by cortico-spinal projections, synergy combinations may be encoded by populations of neurons in the cerebral cortex, especially for complex visuomotor control tasks such as reaching and grasping. In the monkey, muscle activation patterns evoked by electrical stimulation of motor cortical areas can be accurately reconstructed by a small number of muscle synergies highly similar to those extracted during reaching and grasping of objects of different shapes and sizes^56^. Moreover, the activity recorded from a population of cortical units shows spatiotemporal synergies with similar dimensionality, timing, and amplitude modulation to those of the muscle synergies^57^. In particular, such synergies are recruited sequentially, suggesting that the temporal dimension could represent a critical feature to further characterize to better understand sparseness in synergy combinations.

Our results speak also to an ongoing debate on the role of synergies in motor control. According to one view, synergies provide a simplified substrate enabling the control of a variety of movements by means of combination rules^8,16,21^. According to a second view, synergies are task dependent; that is, within the available degrees-of-freedom space, a given task defines a sub-space that is relevant to the task and instead accumulates noise into a redundant sub-space^20,25,60–62^. Our results partially reconcile these views by showing that different types of hand actions can be reconstructed as a simple linear combination of the whole set of atoms. However, specific action classes may use separate sub-spaces (common atoms plus selective atoms), which can overlap, within the space defined by the whole set of atoms. This representational scheme seems to be more sophisticated than previously theorized, possibly exploiting the full power of overcomplete (and redundant) representations that feature prominently in recent machine learning approaches^29,34–39^.

### Novel empirical predictions

**Figure 7:**
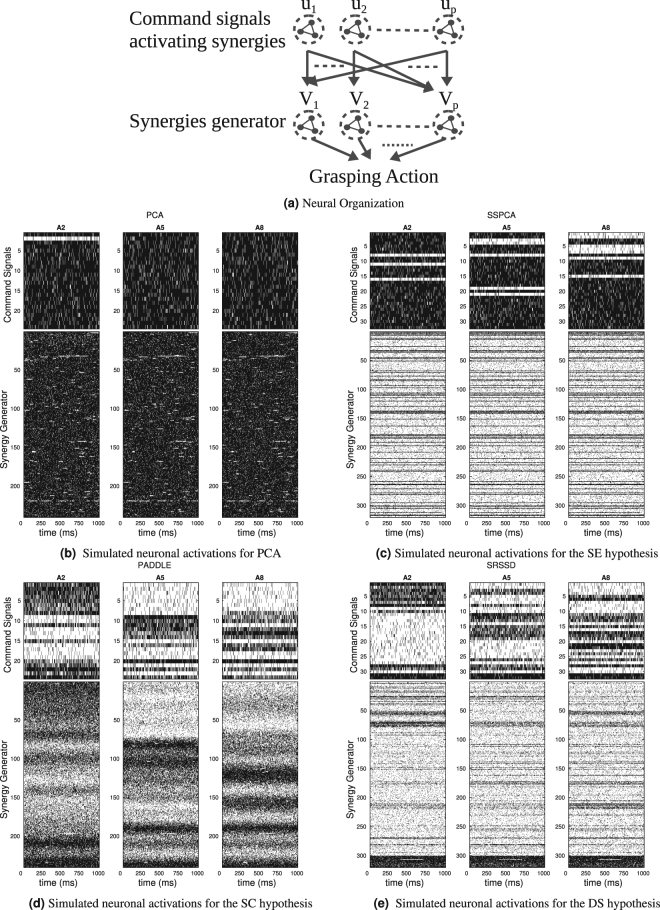
Possible neuronal implementation of synergy-based control under the four computational schemes discussed in this article. Panel (a) shows the putative hierarchical neuronal organization for synergy-based control that we use for the comparison. Here, groups of neurons (within dotted circles) at the higher hierarchical level encode command signals (*u*) and groups of neurons (within dotted circles) at the lower hierarchical level encode synergy pattern generators (*V*). The command signals generated at the higher level activate and modulate synergy pattern generators at the lower hierarchical level, in order to control hand DoFs during grasping actions. Panels (b–e): simulated neuronal activity (raster plots) during grasping actions of 1000 ms under the four different schemes considered in this article. Spikes are in black. See the main text for further details.

The double sparsity hypothesis suggests novel empirical predictions that can be tested experimentally. One such prediction regards the specific neuronal activation patterns that one should expect under the double sparsity (DS) scheme, as opposed to the three other schemes: PCA, sparse combination (SC), sparse elements (SE). There are of course several possible neuronal implementations of synergy based-control; but in order to make a comparison feasible, here we focus on a possible hierarchical neuronal organization for synergy-based control (see Fig. 7) in which the (functional) sparsity in the combination and/or elements directly translates into the (neuronal) sparsity of neuronal populations. This neuronal coding scheme is of course not mandatory–sparsity might be reflected, for example, in the variance of the population neural space^63^, in the patterns of connectivity of the synergy-coding neuronal population with pools of motorneurons rather than in population activity, or in other ways–but is useful here to exemplify the main differences between the four schemes.

The generic hierarchical synergy based-control architecture used for the comparison is shown in Fig. 7a. Within this hierarchical architecture, groups of neurons (within dotted circles) at the higher hierarchical level encode command signals (corresponding to *u* in Equation (1) and groups of neurons (within dotted circles) at the lower hierarchical level encode synergy pattern generators (*V*). The command signals generated at the higher level activate and modulate synergy pattern generators at the lower hierarchical level, in order to control hand DoFs during grasping actions.

Based on this idea, we simulated the neuronal activity patterns (raster plots), at both hierarchical levels, under the four different hypotheses considered in this paper, see Fig. 7b–e. The 32 neurons in the top boxes correspond to the 32 command signals in the simulations. To simulate firing rates at this higher hierarchical level, we converted the usage value (see Equation (9) in Experimental Procedure) of the *k* − *th* Synergy with respect to the *j* − *th* Action Class into a firing probability. Indeed, this usage value corresponds to the command signals that activate the *k* − *th* Synergy during the execution of grasping actions belonging to the *j* − *th* Action Class. Figure 7b–e plot samples from binned probabilities (bins are of 30ms) for the duration of an action (1000ms); each black tick thus corresponds to the fact that a neuron is active during one such bins.

The 320 neurons in the bottom boxes correspond to the Dofs involved in all synergies **V**^*k*^ (10 Dof for 32 synergies). To simulate firing rates at this lower hierarchical level, the Dofs resulting with an absolute value greater than zero during a grasping actions, were converted into the firing probability of the neurons belonging to the group of neurons devoted to generate the k-th synergy. For each class action *A*_*i*_, these firing probabilities were modulated by the usage value of the synergy with respect to the class *A*_*i*_.

Note that for illustrative purposes, we introduced two main simplifications with respect to a more comprehensive biological scheme: first, we only show one neuron for each degree of freedom (for each synergy), whereas the neuronal mapping of degrees of freedom is certainly more sophisticated and might involve populations of neurons. Second, we have simplified the control scheme, omitting (neuronal substrate possibly corresponding to) the fine-grained control of angles and only considering whether or not a synergy (at the higher hierarchical level) or a degree of freedom (at the lower hierarchical level) is used during the action. In this way, neuronal activation at the higher level has no temporal dependency (a synergy is either used or not used) and might correspond to a sort of tonic modulation of the neuronal population that encodes a synergy. Neuronal activation at the lower level is temporally dependent on the usage, or not usage, of a degree of freedom, but not the values that the degree of freedom would assume over time. The results shown in the raster plots are thus meant to exemplify the key differences between the four schemes (under the hypothesized hierarchical synergy-based control architecture), not to map literally to brain activation.

Despite these simplifications, the simulated raster plots (Fig. 7b–e) permit to appreciate the key differences between possible patterns of neuronal activation associated to the four synergy coding schemes. The PCA scheme would predict that, in the absence of any sparsity, all groups of neurons corresponding to both command signals (higher level) and synergy generators (lower level) should show sustained activation over time. The SE hypothesis would predict that all groups of neurons in the higher layer should show sustained activation over time; moreover, the same groups of neurons in the lower layer should be active across all action classes - thus showing a lack of selectivity. The SC hypothesis would predict a lack of selectivity within groups of neurons that correspond to synergy generators; i.e., in the sense that the 32 neurons that correspond to a synergy generator are either all active or all inactive. The DS hypothesis would predict instead that fewer neuronal groups are recruited both at the level of command signals and of synergy generators, coherent with the idea that partially distinct sets of synergies are used for different tasks. Furthermore, even within the same group of neurons corresponding to a single synergy generator, different neurons (corresponding to different Dofs) should be active across different action classes - which is in keeping with a sparse use of Dofs and distinguishes the DS from the SC hypothesis. These specific neural-level predictions of the DS hypothesis remain to be tested in future studies.

## Materials and Methods

### Dataset of grasping actions

We collected a dataset composed of 9 different types of reach-to-grasp actions. To select the actions, we used the classification made in^64^ (see Fig. 2): *Palmar-Pinch*(*A*_1_), *Quadpod*(*A*_2_), *Ring*(*A*_3_), *Lateral*(*A*_4_), *Extension-Type*(*A*_5_), *Parallel*(*A*_6_), *Precision-Sphere*(*A*_7_), *Sphere-4-finger*(*A*_8_) and *Sphere-3-finger*(*A*_9_).

In this classification, *A*_1_, *A*_2_, *A*_6_ and *A*_7_ are *precision-grasp* actions; *A*_3_ and *A*_5_, *A*_8_ and *A*_9_ are *power-grasp* actions; and *A*_4_ is an *intermediate-grasp* action. Figure 2 exemplifies the objects (having different size and shape) that can be considered as usual targets of the actions *A*_1_–*A*_9_, and which we used in our data recordings.

The 9 reach-to grasp actions were recorded by means of the HumanGlove (Humanware S.r.l., Pontedera (Pisa), Italy) endowed with 16 sensors. Wrist related sensors were discarded for this experiment, whereas 10 hand related sensors were considered, following^23^. More specifically, sensors measuring angles of the carpometacarpal (CMC) and metacarpophalangeal (MCP) joints of the thumb and the metacarpophlangeal (MCP) and proximal interphalangeal (PIP) joints of the other four fingers were considered, for a total of *s* = 10 sensors.

Six right-handed subjects (20–30 years old and without neurological disorders) took part in the experiments. A total of 450 grasping actions were recorded for each subject. Each subject performed 50 trials for each type of grasping action *A*_1_–*A*_9_. To avoid fatigue, a rest interval of about 5 seconds was given between trials, and a rest interval of about 2 min was given between different types of grasping actions. Subjects were seated at a table with two clearly visible surface marks (*m*_1_ and *m*_2_) placed at a distance of roughly 40 cm from each other. For each target object, subjects were asked to position their right hand on starting position *m*_1_ and in prone position, and to reach and grasp the target object placed on mark *m*_2_. A computer generated beep was used to signal the start and the end of the action. The interval between the first beep (start) and the second beep (end) amounted to about 2 seconds so that the subject had time enough for achieving the whole grasping action. Once all the actions were recorded, we truncated the recordings in order to preserve only the relevant parts, i.e., the parts in which the hand was actually moving. We sampled each action in order to have the same length *len* (see Supplementary Materials). Note that we used raw-data normalized by linear mapping of each sensor value in the range [−1, 1]. The linear mapping is based on the maximum and minimum angle values. Thus, a grasping action can be expressed as a *p*-dimensional vector ${\bf{x}}\in {{\mathbb{R}}}^{p}$, with *p* = *len* × *s*, composed of a temporal sequence **x** = [**hc**(*t* = 1), **hc**(*t* = 2), ..., **hc**(*t* = *len*)] of *len* hand-joint configurations ${\bf{hc}}({t}_{k})\in {{\mathbb{R}}}^{s}$. Consequently, for each subject we obtained a labelled dataset **X**. Each row **X**_*i*_ is associated with a label specifying the action type. For the sake of clarity, in supplementary materials sample recordings of raw data are shown.

To evaluate the data variability, we performed a principal component analysis on the collected dataset, separately for each subject (see Supplementary Materials). The first 3 components account for about 80% of the variability of the data, and the first 8 components for about 95%. This preliminary analysis lends support for the idea that synergy representation may use a limited set of recurrent elements (and sparsity). Given this, it is also worth noting that in the context of *dictionary learning* approaches, dictionaries composed of more than 10 elements can be considered to be overcomplete.

### Temporal Postural Synergies as a model of hand actions

According to the *Temporal Postural Synergies* (*TPSs*) approach that we adopted in this study, hand actions (expressed here as temporal sequences of hand-joint configurations) should be represented by combinations of TPSs, that is, of specific patterns in the space of hand-joint configurations varying over time^9,15,65^.

More specifically, in this formalism a hand action can be formally described as follows:

1 $${\bf{h}}{\bf{c}}(t)=\sum _{j=1\,}^{r}\sum _{k=1}^{{k}_{j}}{u}_{jk}{{\bf{V}}}^{j}(t-{t}_{jk}).$$

where **hc**(*t*) ∈ *R*^*s*^ is the hand configuration with *s* degrees of freedom at the time *t*, **V**^*j*^(*t*) ∈ *R*^*s*^ is the value at time *t* of the *j*-th element of a pre-defined set of *r* temporal postural synergies, *k*_*j*_ is the number of repeats of the *j*-th synergy used in **hc**(*t*), and *u*_*jk*_ and *t*_*jk*_ represent the weight coefficient and time shift, respectively, of the *k*-th repeat of the synergy **V**^*j*^. At the neurophysiological level, it has been argued that the weights (or coefficients) of TPSs summations may represent signals coming from premotor and motor brain areas which recruit the suitable TPSs, and the TPSs may be encoded by the lower layers of the pyramidal organization of motor representation and control^8,23^.

In our experiments, we took advantage of representing hand actions as linear combinations of TPSs. Indeed, one can suppose that rapid movements such as grasping actions minimize reaction times and constrain the synergies to combine instantaneously^8,23^. In keeping with the hand model expressed in equation (1), a grasping action at time *t* can be represented and controlled by a weighted summation of TPSs without time shifts (see^23^) as expressed in the following equation:

2 $${\bf{h}}{\bf{c}}(t)=\sum _{j=1}^{r}{u}_{j}{{\bf{V}}}^{j}(t)$$

### Dictionary learning approaches

While Principal Component Analysis (PCA)^27^ has been widely used for the study of synergies, interest is growing around alternative approaches that result in powerful signal representations, for example see^29,34–39^. In these *dictionary learning* approaches, signals are represented as linear combinations of a large number of *elements*. The coefficients of the linear combination are obtained using prior information represented by penalization terms or constraints in a minimization problem.

Interestingly, these approaches are informatively grouped into three classes, which (as we will see below) permit to elegantly formulate three competing hypothesis about synergies representation that we test in this study. The first approach can be called *sparse elements*. In this case each element involves just a small number of the original variables (see, for example, Sparse-PCA^34^, sPCA-rSVD^66^ and Structured-Sparse-PCA^41^). The second approach can be called *sparse linear combination of the elements* (or *sparse coding*). Here, an *overcomplete* set of elements is chosen in advance or learned from the data, but the approximation of each signal involves only a restricted number of elements. Hence, signals are represented by sparse linear combinations of the elements (see, for example, MOD^67^, *K* − *SVD*^37^, and ${\ell }_{1}$-regularized^40^). The third approach can be called *double sparsity* and results in a combination of the two former approaches, i.e., a sparse combination of sparse elements; see, for example^42^. It is important to remark that in all these approaches, the orthogonality constraint on elements of PCA is usually violated, the set of elements is usually called *dictionary* (hence the term *dictionary learning*), and each element is called *atom*.

As remarked above, these three dictionary learning approaches seamlessly correspond to three hypotheses on hand action representations–sparse combination hypothesis (SC), sparse elements hypothesis (SE) and double sparsity (DS), respectively–that we compared in this study; see Section 1 for an explanation of the three hypotheses. To model the three hypotheses formally, we used the ${\ell }_{1}$-regularized, Structured-Sparse-PCA (*SSPCA*) and Sparse Representation with Structured Sparse Dictionary (*SRSSD*)^42^ algorithms, respectively.

In the context of these algorithms, the problem that we consider can be described as follows. Let us define a matrix ${\bf{X}}\in {{\mathbb{R}}}^{n\times p}$, where each row corresponds to an experimental observation. One has to find a “good” approximation of **X** as **UV**^*T*^, where ${\bf{V}}\in {{\mathbb{R}}}^{p\times r}$ and ${\bf{U}}\in {{\mathbb{R}}}^{n\times r}$ are two matrices satisfying some predefined constraints. **V** is the *dictionary*, and its columns are the *atoms* (or *dictionary elements*). **U** is the coefficient matrix. The problem to find a good representation of the experimental observations, can be formulated in terms of a minimization problem as expressed in 3 for ${\ell }_{1}$-regularized, in 4 for *SSPCA* and in 5 for *SRSSD*

3 $$\begin{array}{ll}\mathop{{\rm{\min }}}\limits_{{\bf{U}}{\boldsymbol{,}}{\bf{V}}}\frac{1}{2np} & {\Vert {\bf{X}}-{\bf{U}}{{\bf{V}}}^{T}\Vert }_{F}^{2}+\eta \sum _{k=1}^{n}{{\rm{\Omega }}}_{u}({{\bf{U}}}_{k})\\  & s\mathrm{.}t\mathrm{.}\,\forall j,||{{\bf{V}}}^{j}{||}_{2}\le 1\end{array}$$

4 $$\begin{array}{ll}\mathop{{\rm{\min }}}\limits_{{\bf{U}}{\boldsymbol{,}}{\bf{V}}}\frac{1}{2np} & {\Vert {\bf{X}}-{\bf{U}}{{\bf{V}}}^{T}\Vert }_{F}^{2}+\lambda \sum _{k\mathrm{=1}}^{r}{{\rm{\Omega }}}_{v}({{\bf{V}}}^{k})\\  & s\mathrm{.}t\mathrm{.}\,\forall j,||{{\bf{U}}}^{j}{||}_{2}\le 1\end{array}$$

5 $$\begin{array}{ll}\mathop{{\rm{\min }}}\limits_{{\bf{U}}{\boldsymbol{,}}{\bf{V}}}\frac{1}{2np} & {\Vert {\bf{X}}-{\bf{U}}{{\bf{V}}}^{T}\Vert }_{F}^{2}+\lambda \sum _{k\mathrm{=1}}^{r}{{\rm{\Omega }}}_{v}({{\bf{V}}}^{k})\\  & s\mathrm{.}t\mathrm{.}\,\forall j,{{\rm{\Omega }}}_{u}({{\bf{U}}}_{j})\le \eta ,\forall i{\Vert {{\bf{V}}}^{i}\Vert }_{2}=1\end{array}$$

where Ω_*v*_(·) and Ω_*u*_(·) are norms or quasi-norms constraining or regularizing the solutions of the minimization problem, and the parameters *λ* ≥ 0 and *η* ≥ 0 control the extent to which the dictionary and the coefficients are regularized, respectively. The norm ${\ell }_{1}$ is usually chosen to induce sparsity. This norm penalizes solutions containing many coefficients different from zero. For further details see Supplementary Materials.

### Experimental procedure

To adjudicate between three competing hypotheses on action representation–SC, SE and DS–we implemented them in terms of three dictionary learning techniques–${\ell }_{1}$-regularizated, *SSPCA* and *SRSSD*, respectively–and compared these approach between them and against a standard (baseline) action representations using *PCA* in the context of action multi-class classification problems. The idea underlying the comparison is that classification accuracy provides a general metric to evaluate the quality of the algorithms and the associated hypotheses. We hypothesized that the sparse methods would have provided better classification accuracy than PCA, thus supporting the hypothesis that actions can be represented and controlled by 1) switching among different sets of synergies for distinct grasping and manipulation tasks and/or 2) using synergies which use a subset of the possible DoFs (see^68^ for a related approach).

We considered two experimental scenarios. The first scenario is a nine-classes classification problem, whereas the second scenario is a *c*-classes classification problems, with *c* = 2, 4, 6, 8, considering all the possible *c*-ple of class actions, for a total of 255 distinct classification problems (36 2-classes problems, 126 4-classes problems, 84 6-classes problems, and 9 8-classes problems). This latter scenario permits to test another empirical prediction: that the relative importance of sparsity increases as the number of classes increases.

For each subject and for each classification problem we have a labelled dataset **X **∈ *R*^*N*×*p*^ with *p* = *s. len*, where *s* is the number of used data-glove sensors and *len* is the length of each action. **X** is composed of *c* classes with *c *∈ {2, 4, 6, 8, 9}, each one composed of *N* = 50 actions. We have then split this dataset into a training set **X**_*tr*_, a validation **X**_*val*_, and a test set **X**_*te*_, of size *N*_*tr*_ = 0.4*N*, *N*_*val*_ = 0.3*N*, and *N*_*te*_ = 0.3*N*, respectively. A dictionary **V**_*me*,*ps*_ ∈ *R*^*p*×*r*^ is learned on **X**_*tr*_ for each method $me\in \{{\ell }_{1}-regularized,\,SSPCA,\,SRSSD,\,PCA\}$ and for each set of values of the method parameters *pv*. From validation and test set we built noisy validation sets ${{\bf{X}}}_{val}^{\sigma }$, and noisy test sets ${{\bf{X}}}_{te}^{\sigma }$, adding a zero-mean Gaussian noise with *σ* ∈ {0.2, 0.4, 0.6, 0.8}. For each dictionary **V**^*me*,*pv*^ computed on **X**_*tr*_, we applied the method *me* on the validation set ${{\bf{X}}}_{val}^{\sigma }$ for each each set of values of the parameters keeping **V**^*me*,*pv*^ fixed. Thus, for each method *me* and for each set of values *pe* we obtained a representation of the actions in terms of a coefficient matrix ${{\bf{U}}}_{me,ps}\in {R}^{{N}_{val}\times r}$. For each solution, a regularized linear regression model between the action representation and the grasping type was used as multi-class classifier^69^. The regularization parameter for the linear regression model *λ*_*rm*_ was varied in the range *log*_10_(*λ*_*rm*_) ∈ [−20, −19, ..., 1]. The classifier is fed with the action representations expressed in terms of the computed coefficient matrix **U**_*me*,*ps*_. The classifier is trained and evaluated by a 5-fold cross validation approach. The performance of the multi-class classifier is measured by the mean of the accuracies (the number of actions correctly classified over the total number of actions). The parameters of both the methods and the classifier have been selected by taking the ones offering the best performance on the validation set. Then, the methods and the classier have been applied on the test sets ${{\bf{X}}}_{te}^{\sigma }$.

For *SSPCA* and *SRSSD*, we chose a penalization term on the atoms which favors atoms with components corresponding to the same hand-joint angle simultaneously different from zero (see Appendix). This choice is aimed at obtaining grasping action representations as linear combinations of atoms representing the dynamics of a restricted number of hand-joint angles. For example, in order to obtain atoms that describe the temporal evolution of the first hand-joint angle only, the penalization term should favor atoms composed of all zeros except for the values corresponding to the indexes 1, *s* + 1, 2. *s* + 1, and so on^60–73^.

For each solution obtained by ${\ell }_{1}$-regularized, *SSPCA* and *SRSSD* we computed both coefficient sparsity (see equation 6, where **U**_*j*_ are the coefficients of the *j*-th action) and dictionary sparsity (see equation 7, where **V**^*j*^ is the *j*-th atom.). Moreover, for each solution we evaluated both the capability of each atom **V**^*k*^ to represent all the actions of the *c* classes and the capability of each atom to represent just one of the *c* action classes by a commonality and selectivity measure, resepctively. These measures are defined as follows (see^16^):

6 $$coeffSparsitity=1-\frac{1}{nr}\sum _{j}{\Vert {{\bf{U}}}_{j}\Vert }_{0}$$

7 $$dictSparsity=1-\frac{1}{rp}\sum _{j}{\Vert {{\bf{V}}}^{j}\Vert }_{0}$$

8 $$commonality({{\bf{V}}}^{k})=\frac{{M}_{{{\bf{V}}}^{{k}}}}{1+{S}_{{{\bf{V}}}^{{k}}}}$$

9 $$selectivity({{\bf{V}}}^{k},{A}_{i})=usage({{\bf{V}}}^{k},{A}_{i})(1-\frac{1}{C-1}{\sum }_{j\ne i}usage({{\bf{V}}}^{k},{A}_{j}))$$

where ${\Vert \cdot \Vert }_{0}$ is the *l*_0_ norm which counts the number of non zero elements, $usage({{\bf{V}}}^{k},\,{A}_{i})=\frac{1}{card(i)}{\sum }_{j\in {A}_{i}}{\Vert {u}_{jk}\Vert }_{0}$, *card*(*i*) is the number of actions belonging to *A*_*i*_, *C* is the number of action classes, ${M}_{{{\bf{V}}}^{k}}$ and ${S}_{{{\bf{V}}}^{k}}$ are the mean and standard deviation of the *usage* values of **V**^*k*^ over all action types. Note that *commonality* (**V**^*k*^) and *selectivity* (**V**^*k*^, *A*_*i*_) lie between 0 and 1. A commonality value close to 1 means that the corresponding synergy is widely used by almost all the action types. An atom **V**^*k*^ with a high selectivity for a class *C*_*i*_ is mainly used in just this type of action. *selectivity* (**V**^*k*^, *A*_*i*_) assumes the maximum value 1 when **V**^*k*^ is exclusively used by all the actions belonging to *A*_*i*_.

Note that commonality and selectivity values give a deeper comprehension (compared to coefficient sparsity values) of how the actions are represented in terms of atoms. Indeed, coefficient sparsity values measure how many atoms are used for representing all the actions, selectivity values measure to what extent atoms are widely used for *one* or *a few* action classes, while commonality values measure to what extent atoms are widely used for *all* or *many* action classes.

## Electronic supplementary material

Supplementary Materials
