# Supplementary material for: Evidence for sparse synergies in grasping actions
Source: Sci Rep. 2018 Jan 12;8:616. doi: 10.1038/s41598-017-18776-y (PMC5766604; doi:10.1038/s41598-017-18776-y)
Supplement: Supplementary file 1 — Supplementary Materials [file 41598_2017_18776_MOESM1_ESM.pdf]

Supplementary Materials

Evidence for sparse synergies in grasping actions

Roberto Prevete[1,*,+], Francesco Donnarumma[2,+], Andrea d'Avella[3,4,+], Giovanni Pezzulo[2,+]

SSPCA and *SRSSD* Algorithms

The structured sparsity of the dictionary elements in equations 4, 5 is imposed by choosing

$$\Omega_v(\mathbf{V}^k) = \left\{ \sum_{i=1}^{s} \| \mathbf{d}^i \circ \mathbf{V}^k \|_2^{\alpha} \right\}^{\frac{1}{\alpha}} \tag{10}$$

where $\alpha \in (0,1)$, and each $\mathbf{d}^i$ is a p-dimensional vector satisfying the condition $d_j^i \geq 0$, with $i = 1,2,...,s$. The vectors $\mathbf{d}^i$ define the structure of the dictionary elements. More specifically, each $\mathbf{d}^i$ individuates a group of variables corresponding to the set $G^i = \left\{ j \in \{1,\ldots,p\} : d_j^i = 0 \right\}$. The norm $\| \mathbf{d}^i \circ \mathbf{V}^k \|_2^{\alpha}$ penalizes the variables selected by $d_j^i > 0$. Thus, by this norm each vector $\mathbf{d}^i$ induces non zero values for the j-th elements of the atom $\mathbf{V}^k$, when $j \in G$. The resulting set of selected variables depends on the contribution of each $\mathbf{d}^i$ as described in[41,70].

From the work in[41], the problems 4 and 5, considering 10, can be reformulated as follows:

$$\min_{U,V,H} \frac{1}{2np} \|\mathbf{X} - \mathbf{U}\mathbf{V}^T\|_F^2 + \frac{\lambda}{2} \sum_{k=1}^{r} \left[(\mathbf{V}^k)^T Diag(\mathbf{Z}^k)^{-1} \mathbf{V}^k) + \|\mathbf{H}_k\|_{\beta} \right] \\ s.t.\ \forall j,\ \|\mathbf{U}^i\|_2 \leq 1 \tag{11}$$

$$\min_{U,V,H} \frac{1}{2np} \|\mathbf{X} - \mathbf{U}\mathbf{V}^T\|_F^2 + \frac{\lambda}{2} \sum_{k=1}^{r} \left[(\mathbf{V}^k)^T Diag(\mathbf{Z}^k)^{-1} \mathbf{V}^k) + \|\mathbf{H}_k\|_{\beta} \right] \\ s.t.\ \forall j,\ \Omega_u(\mathbf{U}_j) \leq \eta,\ \forall i\ \|\mathbf{V}^i\|_2 = 1 \tag{12}$$

where $\mathbf{H} \in \mathbb{R}_+^{r \times s}$ is a matrix satisfying the condition $h_{ki} \geq 0$, and $\beta = \frac{\alpha}{2-\alpha}$. The matrix $\mathbf{Z} \in \mathbb{R}^{p \times r}$ is defined as $z_{jk} = \left\{ \sum_{i=1}^{s} \left(d_j^i \right)^2 (h_{ki})^{-1} \right\}^{-1}$. Notice that minimizer of 11 and 12 is given in a closed form for fixed both $\mathbf{U}$ and $\mathbf{V}$, and it is equal to $h_{ki} = \bar{h}_{ki} = |y_i^k|^{2-\alpha} \|\mathbf{y}^k\|_{\alpha}^{\alpha-1}$, for $k = 1,2,...,r$ and $i = 1,2,...,s$, where each $\mathbf{y}^k \in \mathbb{R}^{1 \times s}$ is the vector $\mathbf{y}^k = (\|\mathbf{d}^1 \circ \mathbf{V}^k\|_2, \|\mathbf{d}^2 \circ \mathbf{V}^k\|_2, \ldots, \|\mathbf{d}^s \circ \mathbf{V}^k\|_2)$. Moreover, we impose the constraint $\|\mathbf{V}^i\|_2 = 1$ to avoid solutions with $\mathbf{V}$ going towards $\mathbf{0}$, or $\mathbf{U}$ going towards a sparse matrix with elements different from zero having too high values.

In order to solve the problem 12, since the functional in 12 is separately convex in each variable, we follow the usual approach of finding the minimum by alternating optimizations with respect to the values $\mathbf{H}$, to the coefficients $\mathbf{U}$ and to the dictionary $\mathbf{V}$. Thus, *SRSSD* algorithm is an algorithm which has the following three alternate stages[70] (Notice that problem 5 is convex in $\mathbf{U}$ for fixed $\mathbf{V}$ and vice versa):

- ***Update of matrix* H**. In this stage, both $\mathbf{U}$ and $\mathbf{V}$ are assumed fixed and $\mathbf{H}$'s values are updated. As said above, one can update $\mathbf{H}$ by a straightforward equation $h_{ki} = \bar{h}_{ki} = |y_i^k|^{2-\alpha} \|\mathbf{y}^k\|_{\alpha}^{\alpha-1}$, however in order to avoid numerical instability near zero a smoothed update is used as follows: $\mathbf{H}_k \leftarrow max\{\bar{\mathbf{H}}_k, \varepsilon\}$ with $\varepsilon \ll 1$.

- ***Sparse Coding Stage***. The second stage consists in updating $\mathbf{U}$'s values while both $\mathbf{V}$ and $\mathbf{H}$ are fixed. Notice that equation (5) is composed of two terms to be minimized, and the second term does not depend on $\mathbf{U}$. Therefore, the optimization problem posed in (5) can be, in this stage, reformulated as follows: $\min_{\mathbf{U}} \|\mathbf{X} - \mathbf{U}\mathbf{V}^T\|_F^2\ s.t.\ \forall j, \|\mathbf{U}_j\|_0 \leq \eta$. There are a number of well-known "pursuit algorithms" that find an approximate solution for this type of problem (see for example Basis Pursuit (BP)[71] and Orthogonal Matching Pursuit (OMP)[72]). In our approach, a soft thresholding operator[73] is apllied as ℓ_1 norm is used.

- ***Structured Dictionary Element Stage***. The update of dictionary $\mathbf{V}$ is performed in this stage. In this case, as both terms of problem 12 are convex and differentiable terms with respect to $\mathbf{V}$, a closed-form solution for $\mathbf{V}$ can be found, however a proximal method is considered to avoid p matrix inversions:

$$\mathbf{V}^k \leftarrow \Pi_v(Diag(\mathbf{Z}^k) Diag(\|\mathbf{U}^k\|_2^2 \mathbf{Z}^k + np\lambda \mathbf{I})^{-1} \cdot \\ \cdot (\mathbf{X}^T \mathbf{U}^k - \mathbf{V}\mathbf{U}^T \mathbf{U}^k + \|\mathbf{U}^k\|_2^2 \mathbf{V}^k)) \tag{13}$$

  where $\Pi_v(w)$ is simply the Euclidean projection of w onto the unit ball, and the argument of Π_v is obtained by composing a forward gradient descent step on the first term with the proximity operator of the second term of (12).

A description of *SRSSD* approach is given in Algorithm 1. *SSPCA* algorithm is very similar to *SRSSD* algorithm insofar as *Update of matrix H* and *Update of Structured Dictionary* stages are applied only.

Note that when these algorithms are used on actions as those described in this paper, it is possible to use a penalization term on the atoms which favours structured atoms with components corresponding to the same hand-joint angle simultaneously choosing the vectors $\mathbf{d}^i$, with $i = 1,2,...s$, as follows:

$$\begin{aligned} & if\,(j\,mod\,s) == i \\ & \qquad d^i_j = 1; \\ & otherwise \\ & \qquad d^i_j = 0. \end{aligned} \tag{14}$$

where s is the number of used hand-joint angles.

Algorithm 1 *SRSSD* Algorithm

input X
while stop-criterion is not reached
- update $\mathbf{H}$: closed form solution given by $\mathbf{H}_k \leftarrow max\{\bar{\mathbf{H}}_k, \varepsilon\}$ with $\varepsilon \ll 1$.
- sparse coding stage: use OMP (ℓ_0 norm) or soft-thresholding algorithm (ℓ_1 norm) to update $\mathbf{U}$
- dictionary update:
  for $k \leftarrow 1$ **to** r
    $\mathbf{V}^k \leftarrow Diag(\mathbf{Z}^k)Diag(\|\mathbf{U}^k\|_2^2\mathbf{Z}^k + np\lambda\mathbf{I})^{-1}(\mathbf{X}^T\mathbf{U}^k - \mathbf{V}\mathbf{U}^T\mathbf{U}^k + \|\mathbf{U}^k\|_2^2\mathbf{V}^k)$
  endfor
  normalize the columns of $\mathbf{V}$ to have unit norm
endwhile
return $\mathbf{U},\mathbf{V}$

Experimental settings

Raw recordings are given by angles for each finger captured by the HumanGlove. Figure 8 shows a sample recording for Subject 1 and Class A1 with mean and standard deviation of the values of each sensor mapped in the range $[-1,1]$. In order to visualize the whole collected grasping dataset we introduce the notion of *action-type temporal profile*. For each action $\mathbf{hc}(t)$ belonging to one of the 9 types of grasping actions, at a given time t we computed the dot product between the 10-dimensional vector $\mathbf{hc}(t)$ of the sensor values and the unit vector $\mathbf{1}_{10}$. At each time t, the mean of these values over all actions of the same action type were computed. Hence, we obtained 9 different action-type temporal profiles. We have reported the action-type temporal profile for each of the 9 action types in Figure 9.

Figure 10 reports the mean and standard deviation of the cumulative explained variance with respect to the subjects. One can note than the first 3 components account for about 80% of the variability of the data, and the first 8 components for about 95%. Thus, in the context of dictionary learning approaches, dictionaries composed of more than 10 elements can be considered to be overcomplete .

The parameters of the first experiment scenario are reported in Table 1a, those of second experiment scenario Table 1b, where 1) r, the number of atoms used to reconstruct the actions; 2) η, the constraint on the ℓ_1 norm of the rows of $\mathbf{U}$; 3) λ, the regularization parameter for the penalty term on the columns of $\mathbf{V}$, which controls to which extension *SRSSD* and *SSPCA* are constrained to find structured atoms.

For each r value, a PCA decomposition of the actions $\mathbf{X} = \mathbf{U}_{PCA}\mathbf{V}^T_{PCA}$, with $\mathbf{U}_{PCA} \in R^{n\times r}$ and $\mathbf{V}_{PCA} \in R^{p\times r}$, is obtained considering the first r principal components.

Supplementary results and examples

To better understand the functioning of *SRSSD*, Figure 11 shows examples of atoms (from the dictionary computed by this algorithm for subject 1). In particular, Figure 11a shows atoms having the higher value of usage, whereas Figure 11b shows the atoms with the higher value of selectivity for each action class. One can note that atoms selective for each class tend to be more sparse than atom with a high values of usage.

We computed accuracies for each subject, see Table 2, and then to compare subjects we normalized raw accuracies values by computing a t-score for each subject.

We performed five one-way analysis of variance (ANOVAs) to compare the accuracy of the four approaches (*PCA*, ℓ_1, *SSPCA*, and *SRSSD*), with each of the five levels of noise ($\sigma = 0, 0.2, 0.4, 0.6, 0.8$). As shown in Table 3a, we found a significant

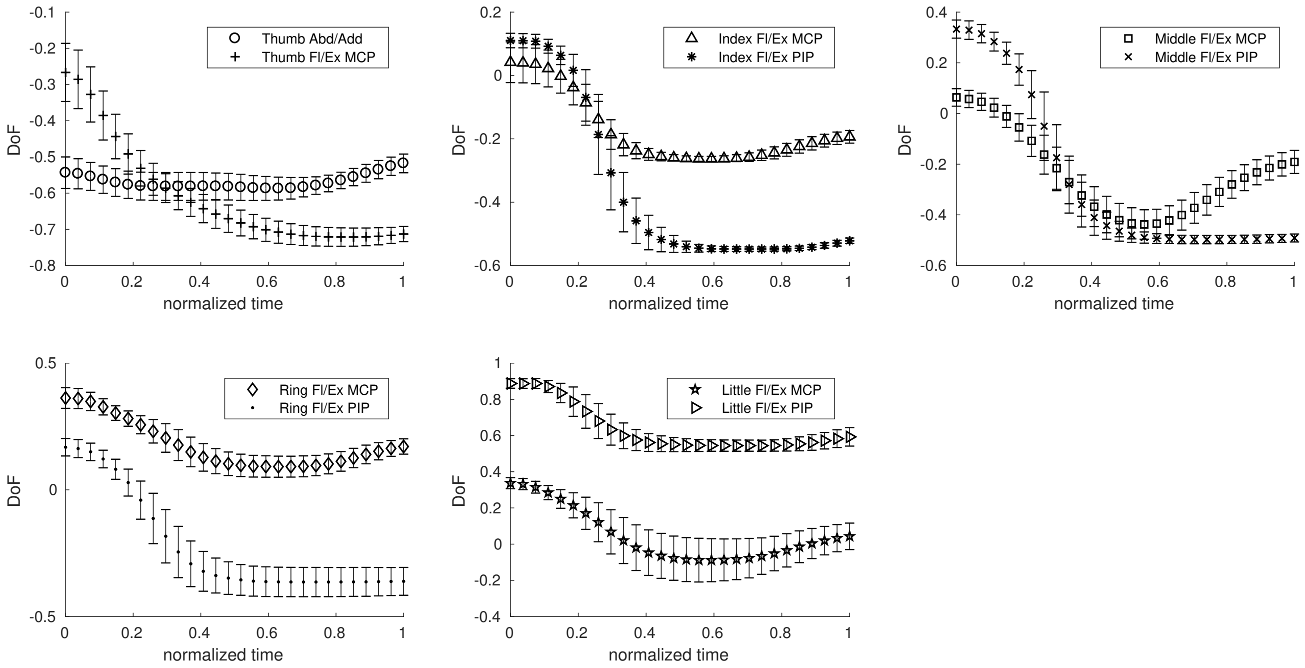

Figure 8. *Raw recording Data.* A sample of recording data for Subject 1, Class A1. The panels show mean and standard deviation of sensor values for each finger.

effect of condition (approaches) in all the five cases. Post-hoc analyses (t-tests) showed that the *SRSSD* approach performs significantly better than all the other approaches, for all the five levels of noise, see Tables 3b-3f.

Similarly to the first scenario, we computed accuracies for each subject, see Table 4, and then to compare subjects we normalized raw accuracies values by computing a t-score for each subject.

We performed a one-way analyses of variance (ANOVAs) to compare the performance of the four approaches (*PCA*, ℓ_1, *SSPCA*, and *SRSSD*), for each of the four different kind of classification problems. As shown in Table 5a, we found a significant effect of condition (approaches) in all the analyses. Post-hoc analyses (t-tests) showed that the *SRSSD* approach performs significantly better than all the other approaches, for all the four cases, see Tables 5b-5e.

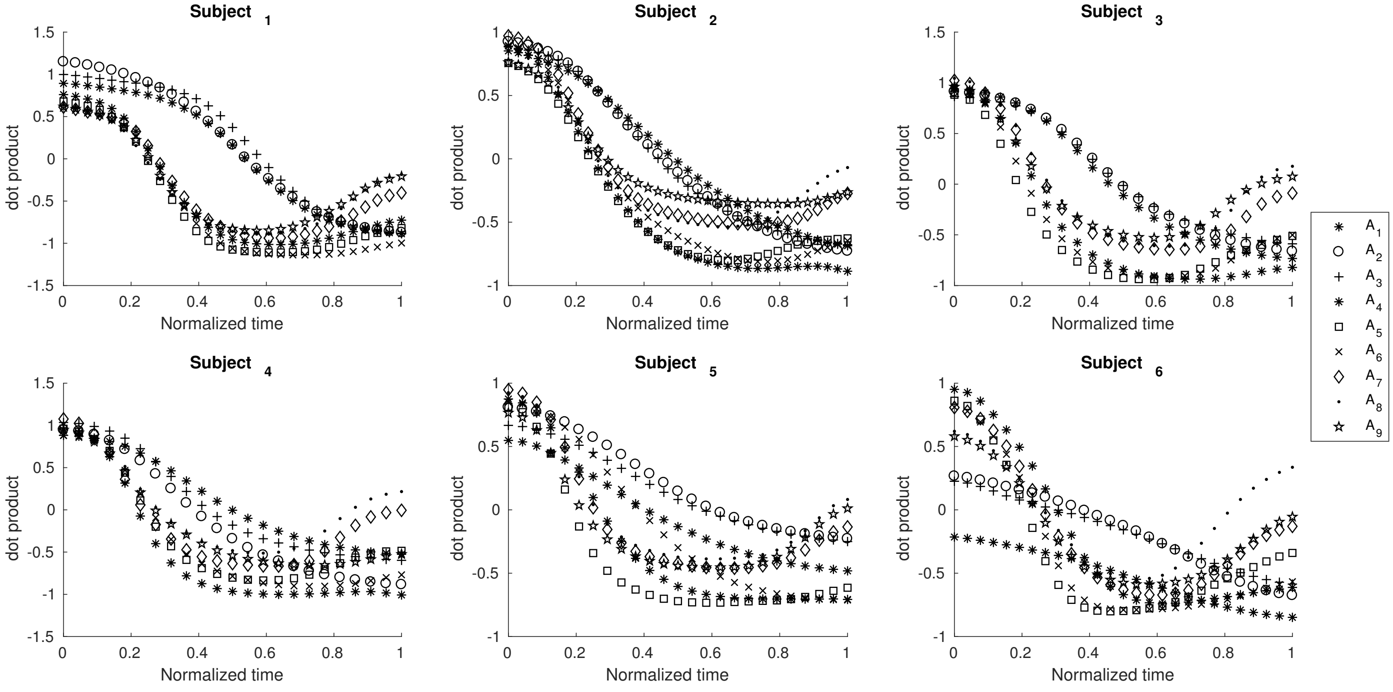

Figure 9. *Recording Data.* The panels show the action-type temporal profile, i.e, the dot product between mean angles for all DoFs and a unity vector for each class at each time. Data for all subjects are shown.

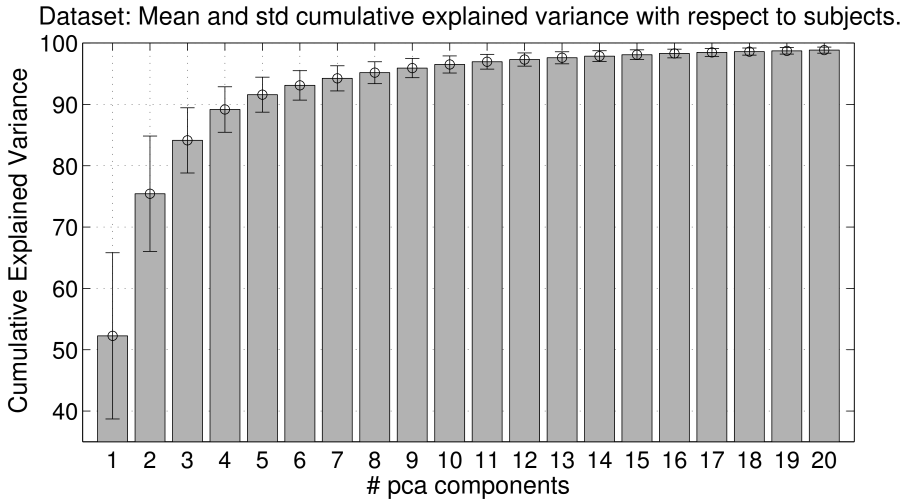

Figure 10. *Cumulative explained Variance, second Scenario*.The Figure shows mean and standard deviation of the mean of cumulative variance with respect to subjects, in the whole dataset.

c (number of action classes)	9
r (number of atoms)	8, 16,24
η (coefficient constraint in *SRSSD* and ℓ_1)	10 equidistant values in $[0.001, 0.1]$
$\log(\lambda)$ (atom constraint in *SRSSD* and *SSPCA*)	10 equidistant values in $[-7, -3]$
len (action length)	37
$log_{10}(\lambda_{rm})$ (classifier regularization parameter)	$[-20, -19, ..., 1]$
σ (noise)	$[0, 0.2, 0.4, 0.6, 0.8]$

(a) Parameters, first scenario

c (number of action classes)	$2, 4, 6, 8$
r (number of atoms)	8, 16,24
η (coefficient constraint in *SRSSD* and ℓ_1)	10 equidistant values in $[0.001, 0.1]$
$\log(\lambda)$ (atom constraint in *SRSSD* and *SSPCA*)	10 equidistant values in $[-7, -3]$
len (action length)	37
$log_{10}(\lambda_{rm})$ (classifier regularization parameter)	$[-20, -19, ..., 1]$
σ (noise)	$[0.8]$

(b) Parameters, second scenario

Table 1. *Experimental parameters*. Panel (a) shows parameters for the nine-classes classification problem, first scenario. Panel (b) shows parameters for the classification problems with $c \in 2, 4, 6, 8$ classes, second scenario.

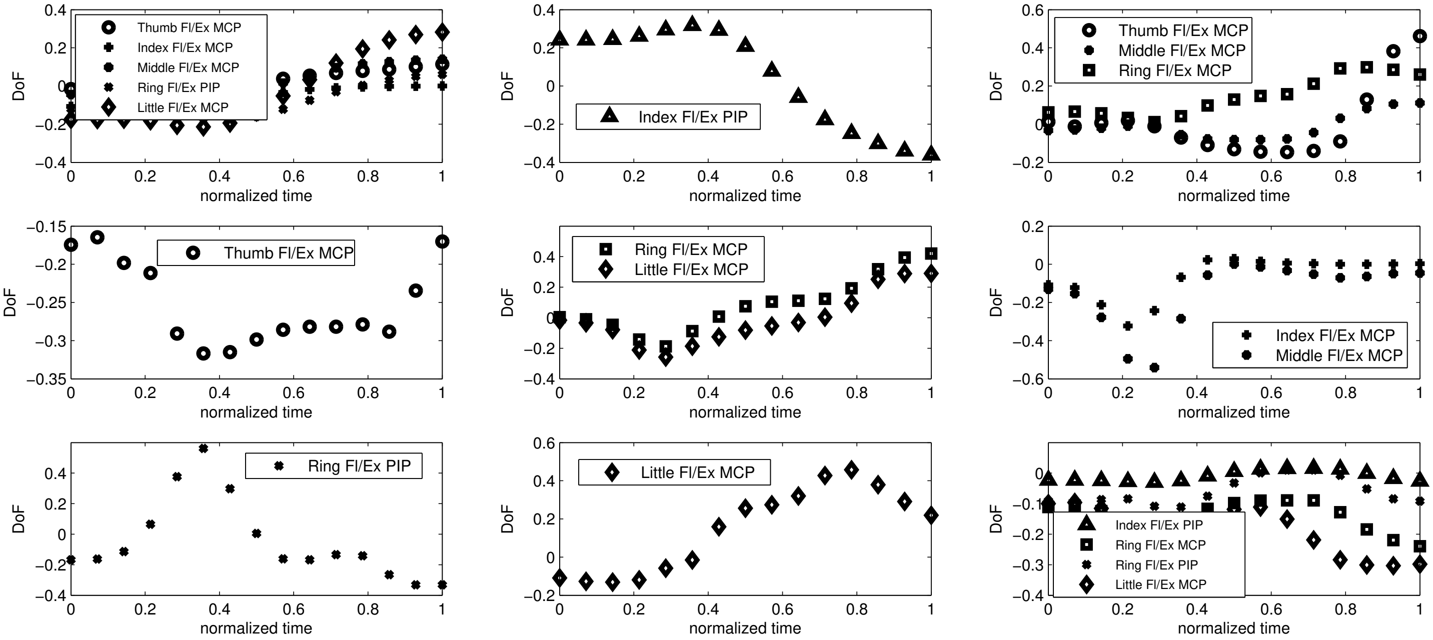

(a) Atoms with the highest value of usage

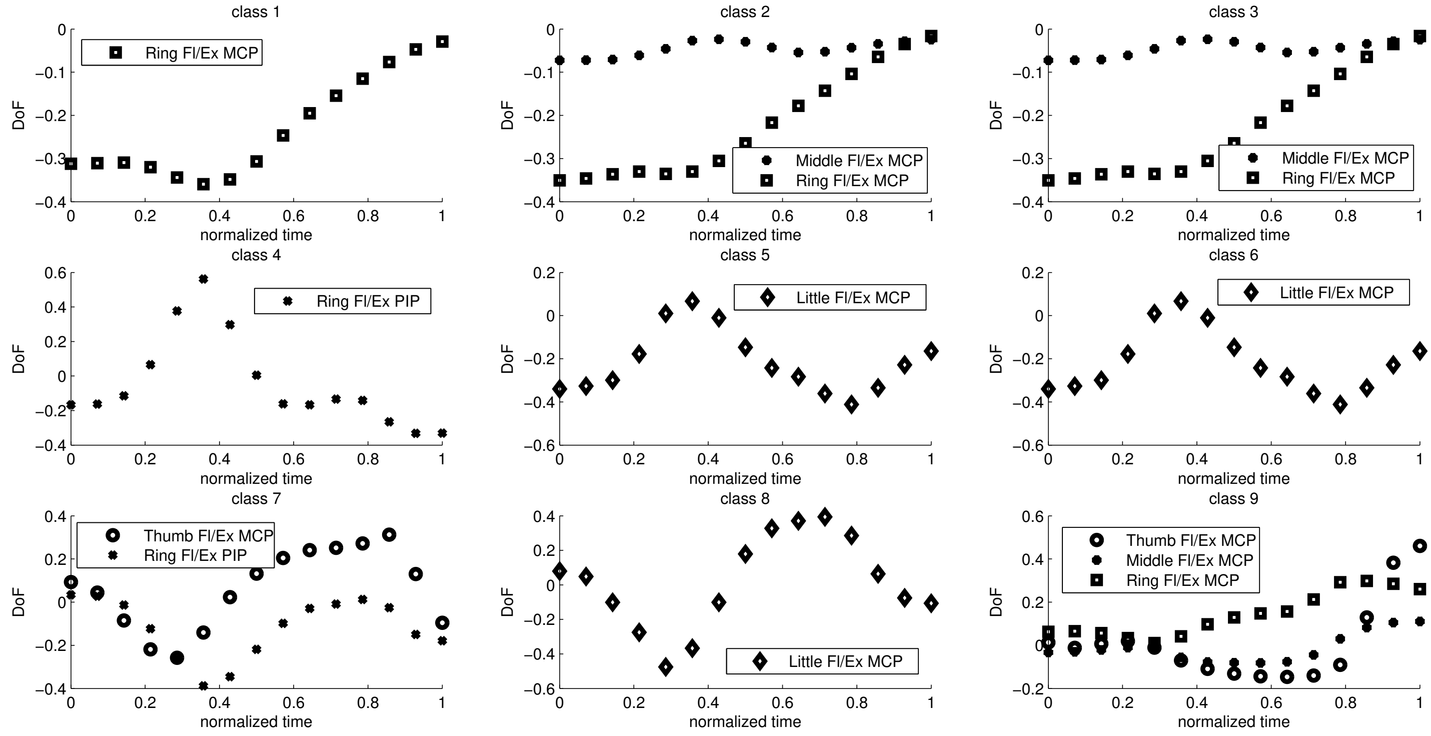

(b) Atoms with the highest value of selectivity

Figure 11. *Dictionary Example*. Example of Dictionary computed by the best (*SRSSD*) algorithm in the multi-classes classification problem of the first Scenario, which includes all the grasping classes. Panel (a) shows the nine atoms with higher values of usage. Panel (b shows the atoms with the highest value of selectivity for each action class.

Subject	Noise	pca	sspca	l1	srssd
Sub 1	$\sigma = 0$	$9.5 \cdot 10^{-01}$	$9.5 \cdot 10^{-01}$	$\mathbf{9.6 \cdot 10^{-01}}$	$\mathbf{9.6 \cdot 10^{-01}}$
Sub 1	$\sigma = 0.2$	$9.2 \cdot 10^{-01}$	$9.2 \cdot 10^{-01}$	$\mathbf{9.4 \cdot 10^{-01}}$	$\mathbf{9.4 \cdot 10^{-01}}$
Sub 1	$\sigma = 0.4$	$8.8 \cdot 10^{-01}$	$9.0 \cdot 10^{-01}$	$9.1 \cdot 10^{-01}$	$\mathbf{9.2 \cdot 10^{-01}}$
Sub 1	$\sigma = 0.6$	$8.4 \cdot 10^{-01}$	$8.7 \cdot 10^{-01}$	$8.6 \cdot 10^{-01}$	$\mathbf{8.8 \cdot 10^{-01}}$
Sub 1	$\sigma = 0.8$	$8.1 \cdot 10^{-01}$	$8.0 \cdot 10^{-01}$	$8.3 \cdot 10^{-01}$	$\mathbf{8.5 \cdot 10^{-01}}$
Sub 2	$\sigma = 0$	$\mathbf{9.7 \cdot 10^{-01}}$	$9.7 \cdot 10^{-01}$	$9.6 \cdot 10^{-01}$	$\mathbf{9.7 \cdot 10^{-01}}$
Sub 2	$\sigma = 0.2$	$9.3 \cdot 10^{-01}$	$9.5 \cdot 10^{-01}$	$9.4 \cdot 10^{-01}$	$\mathbf{9.6 \cdot 10^{-01}}$
Sub 2	$\sigma = 0.4$	$9.1 \cdot 10^{-01}$	$9.1 \cdot 10^{-01}$	$9.0 \cdot 10^{-01}$	$\mathbf{9.2 \cdot 10^{-01}}$
Sub 2	$\sigma = 0.6$	$8.4 \cdot 10^{-01}$	$8.6 \cdot 10^{-01}$	$8.6 \cdot 10^{-01}$	$\mathbf{8.9 \cdot 10^{-01}}$
Sub 2	$\sigma = 0.8$	$8.1 \cdot 10^{-01}$	$8.3 \cdot 10^{-01}$	$8.4 \cdot 10^{-01}$	$\mathbf{8.5 \cdot 10^{-01}}$
Sub 3	$\sigma = 0$	$8.6 \cdot 10^{-01}$	$8.8 \cdot 10^{-01}$	$8.7 \cdot 10^{-01}$	$\mathbf{8.8 \cdot 10^{-01}}$
Sub 3	$\sigma = 0.2$	$8.7 \cdot 10^{-01}$	$8.7 \cdot 10^{-01}$	$8.7 \cdot 10^{-01}$	$\mathbf{8.8 \cdot 10^{-01}}$
Sub 3	$\sigma = 0.4$	$8.3 \cdot 10^{-01}$	$8.4 \cdot 10^{-01}$	$8.4 \cdot 10^{-01}$	$\mathbf{8.5 \cdot 10^{-01}}$
Sub 3	$\sigma = 0.6$	$8.0 \cdot 10^{-01}$	$8.2 \cdot 10^{-01}$	$\mathbf{8.4 \cdot 10^{-01}}$	$8.3 \cdot 10^{-01}$
Sub 3	$\sigma = 0.8$	$8.0 \cdot 10^{-01}$	$8.0 \cdot 10^{-01}$	$\mathbf{8.1 \cdot 10^{-01}}$	$\mathbf{8.1 \cdot 10^{-01}}$
Sub 4	$\sigma = 0$	$9.6 \cdot 10^{-01}$	$9.6 \cdot 10^{-01}$	$9.5 \cdot 10^{-01}$	$\mathbf{9.7 \cdot 10^{-01}}$
Sub 4	$\sigma = 0.2$	$9.2 \cdot 10^{-01}$	$9.4 \cdot 10^{-01}$	$9.2 \cdot 10^{-01}$	$\mathbf{9.5 \cdot 10^{-01}}$
Sub 4	$\sigma = 0.4$	$9.3 \cdot 10^{-01}$	$9.2 \cdot 10^{-01}$	$9.1 \cdot 10^{-01}$	$\mathbf{9.4 \cdot 10^{-01}}$
Sub 4	$\sigma = 0.6$	$8.7 \cdot 10^{-01}$	$8.8 \cdot 10^{-01}$	$8.9 \cdot 10^{-01}$	$\mathbf{9.1 \cdot 10^{-01}}$
Sub 4	$\sigma = 0.8$	$8.3 \cdot 10^{-01}$	$8.3 \cdot 10^{-01}$	$8.4 \cdot 10^{-01}$	$\mathbf{8.5 \cdot 10^{-01}}$
Sub 5	$\sigma = 0$	$9.6 \cdot 10^{-01}$	$\mathbf{9.9 \cdot 10^{-01}}$	$9.7 \cdot 10^{-01}$	$9.8 \cdot 10^{-01}$
Sub 5	$\sigma = 0.2$	$9.6 \cdot 10^{-01}$	$9.7 \cdot 10^{-01}$	$9.7 \cdot 10^{-01}$	$\mathbf{9.9 \cdot 10^{-01}}$
Sub 5	$\sigma = 0.4$	$9.4 \cdot 10^{-01}$	$\mathbf{9.6 \cdot 10^{-01}}$	$9.4 \cdot 10^{-01}$	$9.5 \cdot 10^{-01}$
Sub 5	$\sigma = 0.6$	$9.4 \cdot 10^{-01}$	$9.4 \cdot 10^{-01}$	$9.3 \cdot 10^{-01}$	$\mathbf{9.4 \cdot 10^{-01}}$
Sub 5	$\sigma = 0.8$	$8.7 \cdot 10^{-01}$	$8.8 \cdot 10^{-01}$	$8.7 \cdot 10^{-01}$	$\mathbf{9.0 \cdot 10^{-01}}$
Sub 6	$\sigma = 0$	$9.1 \cdot 10^{-01}$	$9.3 \cdot 10^{-01}$	$9.3 \cdot 10^{-01}$	$\mathbf{9.4 \cdot 10^{-01}}$
Sub 6	$\sigma = 0.2$	$9.0 \cdot 10^{-01}$	$9.2 \cdot 10^{-01}$	$9.2 \cdot 10^{-01}$	$\mathbf{9.3 \cdot 10^{-01}}$
Sub 6	$\sigma = 0.4$	$8.8 \cdot 10^{-01}$	$\mathbf{9.1 \cdot 10^{-01}}$	$8.8 \cdot 10^{-01}$	$\mathbf{9.1 \cdot 10^{-01}}$
Sub 6	$\sigma = 0.6$	$8.2 \cdot 10^{-01}$	$8.5 \cdot 10^{-01}$	$8.5 \cdot 10^{-01}$	$\mathbf{8.6 \cdot 10^{-01}}$
Sub 6	$\sigma = 0.8$	$7.9 \cdot 10^{-01}$	$8.3 \cdot 10^{-01}$	$8.0 \cdot 10^{-01}$	$\mathbf{8.4 \cdot 10^{-01}}$

Table 2. Mean accuracies for each subject obtained on noised ($\sigma \in \{0, 0.2, 0.4, 0.6, 0.8\}$)

Noise	Anova Results	p
0	F[3,20]=7.42	$\mathbf{1.6 \cdot 10^{-03}}$
0.2	F[3,20]=26.82	$\mathbf{3.2 \cdot 10^{-07}}$
0.4	F[3,20]=9.50	$\mathbf{4.2 \cdot 10^{-04}}$
0.6	F[3,20]=17.48	$\mathbf{8.2 \cdot 10^{-06}}$
0.8	F[3,20]=19.85	$\mathbf{3.3 \cdot 10^{-06}}$

(a) ANOVAs

	sspca	l1	srssd
pca	$\mathbf{3.1 \cdot 10^{-02}}$	0.54	$\mathbf{1.1 \cdot 10^{-03}}$
sspca		0.10	$\mathbf{3.2 \cdot 10^{-02}}$
l1			$\mathbf{3.2 \cdot 10^{-03}}$

(b) T-tests, noise: $\sigma = 0$

	sspca	l1	srssd
pca	$\mathbf{5.7 \cdot 10^{-04}}$	$\mathbf{8.1 \cdot 10^{-03}}$	$\mathbf{1.4 \cdot 10^{-07}}$
sspca		0.38	$\mathbf{9.0 \cdot 10^{-04}}$
l1			$\mathbf{4.9 \cdot 10^{-04}}$

(c) T-tests, noise: $\sigma = 0.2$

	sspca	l1	srssd
pca	$\mathbf{4.0 \cdot 10^{-02}}$	0.99	$\mathbf{6.9 \cdot 10^{-04}}$
sspca		$\mathbf{3.5 \cdot 10^{-02}}$	$\mathbf{4.7 \cdot 10^{-02}}$
l1			$\mathbf{4.8 \cdot 10^{-04}}$

(d) T-tests, noise: $\sigma = 0.4$

	sspca	l1	srssd
pca	$\mathbf{1.4 \cdot 10^{-03}}$	$\mathbf{2.4 \cdot 10^{-02}}$	$\mathbf{9.1 \cdot 10^{-06}}$
sspca		0.70	$\mathbf{3.4 \cdot 10^{-04}}$
l1			$\mathbf{5.9 \cdot 10^{-03}}$

(e) T-tests, noise: $\sigma = 0.6$

	sspca	l1	srssd
pca	0.24	$\mathbf{1.2 \cdot 10^{-02}}$	$\mathbf{3.2 \cdot 10^{-07}}$
sspca		0.15	$\mathbf{8.0 \cdot 10^{-05}}$
l1			$\mathbf{2.5 \cdot 10^{-03}}$

(f) T-tests, noise: $\sigma = 0.8$

Table 3. *ANOVA and post-hoc statistical analysis, first scenario.* Panel a shows five one-way ANOVAs, one for each level of noise, comparing the four approaches. Panels (b), (c), (d), (e), (f) show the t-tests comparing the four methods, under different levels of noise, respectively $0, 0.2, 0.4, 0.6, 0.8$. In all panels, significant results are in bold. See the main text for explanation.

Subject	Class	pca	sspca	l1	srssd
Sub 1	2	$1.0 \cdot 10^{+00}$	$1.0 \cdot 10^{+00}$	$1.0 \cdot 10^{+00}$	$\mathbf{1.0 \cdot 10^{+00}}$
Sub 1	4	$9.7 \cdot 10^{-01}$	$9.8 \cdot 10^{-01}$	$9.7 \cdot 10^{-01}$	$\mathbf{9.9 \cdot 10^{-01}}$
Sub 1	6	$9.0 \cdot 10^{-01}$	$9.1 \cdot 10^{-01}$	$9.0 \cdot 10^{-01}$	$\mathbf{9.2 \cdot 10^{-01}}$
Sub 1	8	$8.4 \cdot 10^{-01}$	$8.5 \cdot 10^{-01}$	$8.5 \cdot 10^{-01}$	$\mathbf{8.8 \cdot 10^{-01}}$
Sub 2	2	$1.0 \cdot 10^{+00}$	$1.0 \cdot 10^{+00}$	$1.0 \cdot 10^{+00}$	$\mathbf{1.0 \cdot 10^{+00}}$
Sub 2	4	$9.6 \cdot 10^{-01}$	$9.7 \cdot 10^{-01}$	$9.6 \cdot 10^{-01}$	$\mathbf{9.9 \cdot 10^{-01}}$
Sub 2	6	$8.8 \cdot 10^{-01}$	$8.9 \cdot 10^{-01}$	$8.9 \cdot 10^{-01}$	$\mathbf{9.1 \cdot 10^{-01}}$
Sub 2	8	$8.3 \cdot 10^{-01}$	$8.3 \cdot 10^{-01}$	$8.5 \cdot 10^{-01}$	$\mathbf{8.6 \cdot 10^{-01}}$
Sub 3	2	$1.0 \cdot 10^{+00}$	$1.0 \cdot 10^{+00}$	$9.9 \cdot 10^{-01}$	$\mathbf{1.0 \cdot 10^{+00}}$
Sub 3	4	$9.3 \cdot 10^{-01}$	$9.4 \cdot 10^{-01}$	$9.4 \cdot 10^{-01}$	$\mathbf{9.7 \cdot 10^{-01}}$
Sub 3	6	$8.7 \cdot 10^{-01}$	$8.8 \cdot 10^{-01}$	$8.8 \cdot 10^{-01}$	$\mathbf{9.0 \cdot 10^{-01}}$
Sub 3	8	$8.0 \cdot 10^{-01}$	$8.3 \cdot 10^{-01}$	$8.3 \cdot 10^{-01}$	$\mathbf{8.4 \cdot 10^{-01}}$
Sub 4	2	$1.0 \cdot 10^{+00}$	$1.0 \cdot 10^{+00}$	$1.0 \cdot 10^{+00}$	$\mathbf{1.0 \cdot 10^{+00}}$
Sub 4	4	$9.8 \cdot 10^{-01}$	$9.8 \cdot 10^{-01}$	$9.7 \cdot 10^{-01}$	$\mathbf{1.0 \cdot 10^{+00}}$
Sub 4	6	$9.1 \cdot 10^{-01}$	$9.2 \cdot 10^{-01}$	$9.2 \cdot 10^{-01}$	$\mathbf{9.3 \cdot 10^{-01}}$
Sub 4	8	$8.6 \cdot 10^{-01}$	$8.6 \cdot 10^{-01}$	$8.6 \cdot 10^{-01}$	$\mathbf{8.8 \cdot 10^{-01}}$
Sub 5	2	$1.0 \cdot 10^{+00}$	$1.0 \cdot 10^{+00}$	$1.0 \cdot 10^{+00}$	$\mathbf{1.0 \cdot 10^{+00}}$
Sub 5	4	$9.8 \cdot 10^{-01}$	$9.9 \cdot 10^{-01}$	$9.8 \cdot 10^{-01}$	$\mathbf{1.0 \cdot 10^{+00}}$
Sub 5	6	$9.4 \cdot 10^{-01}$	$9.5 \cdot 10^{-01}$	$9.4 \cdot 10^{-01}$	$\mathbf{9.5 \cdot 10^{-01}}$
Sub 5	8	$9.1 \cdot 10^{-01}$	$9.1 \cdot 10^{-01}$	$9.1 \cdot 10^{-01}$	$\mathbf{9.3 \cdot 10^{-01}}$
Sub 6	2	$1.0 \cdot 10^{+00}$	$1.0 \cdot 10^{+00}$	$1.0 \cdot 10^{+00}$	$\mathbf{1.0 \cdot 10^{+00}}$
Sub 6	4	$9.7 \cdot 10^{-01}$	$9.7 \cdot 10^{-01}$	$9.6 \cdot 10^{-01}$	$\mathbf{9.9 \cdot 10^{-01}}$
Sub 6	6	$8.8 \cdot 10^{-01}$	$9.0 \cdot 10^{-01}$	$9.0 \cdot 10^{-01}$	$\mathbf{9.2 \cdot 10^{-01}}$
Sub 6	8	$8.4 \cdot 10^{-01}$	$8.4 \cdot 10^{-01}$	$8.5 \cdot 10^{-01}$	$\mathbf{8.6 \cdot 10^{-01}}$

Table 4. Mean accuracies for each subject computed for each class. (Noise: $\sigma = 0.8$)

Classes	Anova Results	p
2	F[3,20]=90.77	$\mathbf{8.0 \cdot 10^{-12}}$
4	F[3,20]=369.31	$\mathbf{1.1 \cdot 10^{-17}}$
6	F[3,20]=146.66	$\mathbf{8.8 \cdot 10^{-14}}$
8	F[3,20]=66.68	$\mathbf{1.4 \cdot 10^{-10}}$

(a) ANOVAs

	sspca	l1	srssd
pca	$\mathbf{2.0 \cdot 10^{-03}}$	$\mathbf{2.1 \cdot 10^{-05}}$	$\mathbf{9.8 \cdot 10^{-06}}$
sspca		$\mathbf{2.1 \cdot 10^{-07}}$	$\mathbf{7.2 \cdot 10^{-03}}$
l1			$\mathbf{1.9 \cdot 10^{-09}}$

(b) T-tests, two Classes

	sspca	l1	srssd
pca	$\mathbf{7.4 \cdot 10^{-07}}$	0.50	$\mathbf{2.0 \cdot 10^{-11}}$
sspca		$\mathbf{3.5 \cdot 10^{-06}}$	$\mathbf{2.7 \cdot 10^{-12}}$
l1			$\mathbf{7.0 \cdot 10^{-11}}$

(c) Four Classes

	sspca	l1	srssd
pca	$\mathbf{4.3 \cdot 10^{-05}}$	$\mathbf{3.0 \cdot 10^{-06}}$	$\mathbf{1.6 \cdot 10^{-11}}$
sspca		0.15	$\mathbf{2.0 \cdot 10^{-06}}$
l1			$\mathbf{8.6 \cdot 10^{-10}}$

(d) Six Classes

	sspca	l1	srssd
pca	$\mathbf{3.2 \cdot 10^{-03}}$	$\mathbf{5.9 \cdot 10^{-04}}$	$\mathbf{2.9 \cdot 10^{-09}}$
sspca		0.30	$\mathbf{1.0 \cdot 10^{-06}}$
l1			$\mathbf{6.0 \cdot 10^{-06}}$

(e) Eight Classes

Table 5. *ANOVA and post-hoc statistical analysis, second scenario*. Panel (a) shows four one-way ANOVAs (one for each of the four action classes we considered) comparing the four approaches. Panels (b), (c), (d), (e) show t-tests on different action classes. Significant results are in bold. In all panels, significant results are in bold. See the main text for explanation.
